# Report from the 9th International Symposium on Auriculotherapy Held in Singapore, 10–12 August 2017 †

**DOI:** 10.3390/medicines4030046

**Published:** 2017-06-26

**Authors:** Im Quah-Smith, Gerhard Litscher, Peijing Rong, Terry Oleson, Gary Stanton, Arnyce Pock, Richard Niemtzow, Steven Aung, Raphael Nogier

**Affiliations:** 1 Research Associate RHW, UNSW, Randwick, Australia Director, RWG, 2069 Roseville, Australia; 2 Research Associate CHeBA, University of New South Wales, 2031 Sydney, Australia; 3 Head of the TCM (Traditional Chinese Medicine) Research Center Graz, of the Research Unit of Biomedical Engineering in Anesthesia and Intensive Care Medicine, and of the Research Unit for Complementary and Integrative Laser Medicine, Medical University of Graz, 8036 Graz, Austria; 4 Institute of Acupuncture and Moxibustion, China Academy of Chinese Medical Sciences, Beijing 100700, China; drrongpj@163.com; 5 Ryokan College, Los Angeles, CA 90066, USA; terry.oleson@gmail.com; 6 Emerson Hospital, Concord, MA 01742, USA; gstanton@emersonhosp.org; 7 Uniformed Services University of the Health Sciences (USUHS), Bethesda, MD 20814, USA; arnyce.pock@usuhs.edu; 8 Integrative Medicine Consultant to the United States Air Force (USAF) Surgeon General; Director, USAF Acupuncture & Integrative Med Center, Joint Base Andrews, MD 20762, USA; n5evmd@gmail.com; 9 Faculty of Medicine and Dentistry, Faculties of Extension, Pharmacy & Pharmaceutical Sciences and Rehabilitation Medicine and School of Public Health, University of Alberta, Edmonton, AB T6G 2R3, Canada; draung@aung.com; 10 G.L.E.M. Lyon Medical Studies Group, 49 Rue Merciere, 69002 Lyon, France; nogierr@club-internet.fr

## 1. Preface

Auricular interventions also known as auriculotherapy, auricular medicine and ear acupuncture depending on practice locale, has come of age and has gained the attention of the wider medical community in recent years.

Although the World Health Organisation has, since 1997, accepted that auriculotherapy (AT) has a place in clinical care, it has mostly been utilized in Europe, China and the USA. Until recently, many countries were often not aware of the place of auriculotherapy in clinical diagnosis and therapeutics. 

Much attention has been paid to this discipline in the last few years when Dr. (retired U.S. Air Force Colonel) Richard Niemtzow developed an acute pain management auricular protocol for those sustaining injury in the course of battle that he aptly named Battlefield Acupuncture or BFA. Since then, it has been successfully adopted in other scenarios (emergency rooms, neonatal intensive care, primary care) to reduce pain and trauma, emotional and physical pain. 

Auriculotherapy augments diagnostics and therapeutics in medicine. 

The Vagus and Trigeminal Nerves are represented on the ear and their links to psycho-neuro-endocrino-immuno interactions (PNEI) are now better understood. This connectivity has been shown to be integral to this highly effective approach in clinical care. 

Ease of application and innovative modalities include magnets, seeds and acupressure, low intensity laser light, cautery, microcurrent and cryotherapy.

Even if one does not practise the therapeutics, the finely tuned diagnostic capabilities of auriculotherapy is worthy of one’s time and the effort to learn from some of the best clinicians and researchers in the world at this important symposium.

For the clinicians wishing to include it in their practice or to further augment their knowledge, the symposium will be highly rewarding.

We celebrate the diversity and multi-national input which have culminated in the development of this important symposium. Although the Symposium Book includes printed copies of the original abstracts, authors who wish to have a full paper published in a special issue of “**Auricular Medicine: Gateway to the Brain in Healing**” by **Medicines** MDPI will need to comply with the journal's formal instructions for authors, to include the standard language requirements.

## 2. Keynote Lectures

### 2.1. Innovative Research on Auricular Medicine—Opening Lecture

#### Litscher, G.

Treatment by auricular therapy has a long history. Ear acupoint research has been advancing step by step worldwide. Within this lecture new developments and results from innovative research on auricular medicine will be presented [1–17]. The introduction of lasers into medicine brought besides the already existing stimulation with needles, electricity, pressure and liquids an additional technique to auricular acupuncture. The latest scientific findings on auricular acupuncture with laser (infrared, red, blue, green and yellow) will be discussed in context to the evidence to clinical applications. Furthermore a new system for ear vibration stimulation and the resulting acute effects of vibration and manual ear acupressure on heart rate, heart rate variability, pulse wave velocity, and the augmentation index using new noninvasive recording methods will also be shown.

The Nogier reflex or reflex auriculo-cardiac (RAC; also vascular autonomic signal) is an important method in auricular medicine. New methodological approaches for the detection and quantification of the RAC from the Medical University of Graz will be demonstrated. A new high-resolution imaging technique for the registration of pulsatory surface changes might allow the RAC to be quantified reproducibly for the first time. The methods combine innovative microscope systems (Medical University of Graz), video analysis software, special image processing software (Beijing University of Science and Technology) and visualization of biologically active ear points (Universities from Novosibirsk). Even small, pulse-dependent alterations of the skin surface can be clearly visualized. 

Sino-European transcontinental basic and clinical high-tech auricular acupuncture studies demonstrate the modernization of auricular acupuncture and the scientific way from auricular therapy to auricular medicine.

The studies were supported by the Austrian Ministry of Science, Research and Economy (BMWFW) and the German Academy of Acupuncture (DAA).

1. Litscher, D.; Litscher, G. The history of liquid ear acupuncture and the current scientific state of the art. *J. Pharmacopunct.* **2016**, *19*, 109–113.
2. Litscher, G.; Rong, P.J. Auricular acupuncture. *Evid. Based Complement. Altern. Med*. **2016**, *2016*, 4231260.
3. Rong, P.J.; Zhao, J.J.; Li, Y.Q.; Litscher, D.; Li, S.Y.; Gaischek, I.; Zhai, X.; Wang, L.; Luo, M.; Litscher, G. Auricular acupuncture and biomedical research—A promising Sino-Austrian research cooperation. *Chin. J. Integr. Med*. **2015**, *21*, 887–894.
4. Navrotsky, L.G.; Blokhin, A.A.; Belavskaya, S.V.; Lisitsyna, L.I.; Lyutkevich, A.A.; Poteryaeva, E.L.; Yudin, V.I.; Litscher, G. Patterns of skin luminescence resulting from the visualization of active acupuncture points using optical stimulation. *Integr. Med. Int*. **2015**, *2*, 1–8.
5. Litscher, G.; Bahr, F.; Litscher, D.; Min, L.Q.; Rong, P.J. A new method in auricular medicine for the investigation of the Nogier reflex. *Integr. Med. Int.* **2014**, *1*, 205–210.
6. Shi, X.; Litscher, G.; Wang, H.; Wang, L.; Zhao, Z.; Litscher, D.; Tao, J.; Gaischek, I.; Sheng, Z. Continuous auricular electroacupuncture can significantly improve heart rate variability and clinical scores in patients with depression: First results from a transcontinental study. *Evid. Based Complement. Altern. Med*. **2013**, *2013*, 894096.
7. Round, R.; Litscher, G.; Bahr, F. Auricular acupuncture with laser. *Evid. Based Complement. Altern. Med*. **2013**, *2013*, 984763.
8. Raith, W.; Litscher, G.; Müller, W.; Urlesberger, B. Laseracupuncture—A possible alternative treatment for agitation and pain in neonates? *Paediatr. Anaesth*. **2013**, *23*, 205–206.
9. Gao, X.Y.; Litscher, G.; Liu, K.; Zhu, B. Sino-European transcontinental basic and clinical high-tech acupuncture studies—Part 3: Violet laser stimulation in anesthetized rats. *Evid. Based Complement. Altern. Med*. **2012**, *2012*, 402590.
10. He, W.; Rong, P.J.; Li, L.; Ben, H.; Zhu, B.; Litscher, G. Auricular acupuncture may suppress epileptic seizures via activating the parasympathetic nervous system: A hypothesis based on innovative methods. *Evid. Based Complement. Altern. Med.* **2012**, *2012*, 615476.
11. Gao, X.Y.; Liu, K.; Zhu, B.; Litscher, G. Sino-European transcontinental basic and clinical high-tech acupuncture studies—Part 1: Auricular acupuncture increases heart rate variability in anesthetized rats. *Evid. Based Complement. Altern. Med*. **2012**, *2012*, 817378.
12. Gao, X.Y.; Wang, L.; Gaischek, I.; Michenthaler, Y.; Zhu, B.; Litscher, G. Brain-modulated effects of auricular acupressure on the regulation of autonomic function in healthy volunteers. *Evid. Based Complement. Altern. Med*. **2012**, *2012*, 714391.
13. Litscher, G.; Bauernfeind, G.; Gao, X.Y.; Müller-Putz, G.; Wang, L.; Anderle, W.; Gaischek, I.; Litscher, D.; Neuper, C.; Niemtzow, R.C. Battlefield acupuncture and near-infrared spectroscopy—Miniaturized computer-triggered electrical stimulation of battlefield ear acupuncture points and 50-channel near-infrared spectroscopic mapping. Medical Acupuncture. **2011**, *23*, 263–270.
14. Niemtzow, R.C.; Litscher, G.; Burns, S.M.; Helms, J.M. Battlefield acupuncture: Update. *Med. Acupunct*. **2009**, *21*, 43–46.
15. Litscher, G. Modernization of traditional acupuncture using multimodal computer-based high-tech methods-recent results of blue laser and teleacupuncture from the Medical University of Graz. *J. Acupunct. Meridian Stud*. **2009**, *2*, 202–209.
16. Széles, J.C.; Litscher, G. Objectivation of cerebral effects with a new continuous electrical auricular stimulation technique for pain management. *Neurol. Res*. **2004**, *26*, 797–800.
17. Litscher, G. Computer-based quantification of traditional Chinese-, ear- and Korean hand acupuncture: Needle-induced changes of regional cerebral blood flow velocity. *Neurol. Res*. **2002**, *24*, 377–380.

### 2.2. Mechanisms Underlying the Regulation of Impaired Glucose Tolerance by Auricular Concha Electro-Acupuncture

#### Rong, P.J.

Impaired Glucose Tolerance (IGT) is an abnormal metabolic state between glucose metabolic steady state and diabetes. With an underlying mechanism of insulin resistance and pancreatic β cell dysfunction, it is an important stage during the progression of diabetes. IGT is one of the diseases that shows significant beneficial response to acupuncture treatment (Xiong et al. 2015). Our studies shows that transcutaneous auricular vagus nerve stimulation (taVNS) which is innervated by vagus nerve, would enhance the activity of pancreatic β-cells, promote the secretion of insulin, upregulate the expression of insulin receptors in central as well as peripheral tissues (Li et al. 2014; Li et al. 2014), thus improve glycometabolism. In this study, we would illuminated the mechanism of taVNS at “yidan-pi” auricular acupoints on the regulation of glucose metabolism, its improvement of the IGT state in rat model, as well as its regulation effect on insulin receptor expression and insulin resistance. With emphasize on the influence on the concentrations of glucose and HbA1c, in 100 participants, compared with the sham group, we found that patients receiving taVNS significantly differed in measures of FBG and HbAlc over the course of the 12 week treatment period which suggested that taVNS is a promising, simple, and cost-effective treatment for IGT with only slight risk of mild side-effects.

The studies were supported by the National Natural Science Foundation of China (81674072).

1. Xiong, X.Q.; Wang, A.M. Clinical Research Progress of Auricular Points to Treat with Type 2 Diebetes Mellitus. *Nurs. J. Chin. People’s Lib. Army* **2015**, *32*, 33–35.
2. Li, S.Y.; Zhai, X.; Rong, P.J.; McCabe, M.F.; Wang, X.; Zhao, J.; Ben, H.; Wang, S. Therapeutic Effect of Vagus Nerve Stimulation on Depressive-like Behavior, Hyperglycemia and Insulin Receptor Expression in Zucker Fatty Rats. *PLoS ONE* **2014**, *9*, e112066.
3. Li, S.Y.; Zhai, X.; Rong, P.J.; McCabe, M.F.; Zhao, J.; Ben, H.; Wang, X.; Wang, S. Transcutaneous Auricular Vagus Nerve Stimulation Triggers Melatonin Secretion and is Antidepressive in Zucker Diabetic Fatty Rats. *PLoS ONE* **2014**, *9*, e111100.

### 2.3. Anatomo-Physiological Basis for Auricular Stimulation

#### Deriu, F.

The stimulation of cranial nerves modulates central nervous system (CNS) activity via the extensive connections of their brainstem nuclei to higher order structures. The clinical experience with vagal nerve stimulation (VNS) demonstrates that it exerts robust therapeutic effects, posing however concerns related to its invasiveness and side effects. Trigeminal nerve stimulation (TNS) has been recently proposed as a valid alternative to VNS. The ear presents afferent vagus and trigeminal nerve distribution, then its innervation is the theoretical basis of different reflex therapies, included auriculotherapy. An increasing amount of studies are showing that several therapeutic effects induced by invasive VNS and TNS, can be reproduced by non-invasive auricular nerve stimulation. However, the sites and neurobiological mechanisms by which VNS and TNS exert their therapeutic effects are not clear yet. Accumulating evidence suggest that they share multiple levels of action in the CNS.

### 2.4. Auriculotherapy in Paediatric Neurology: A Promising Tool for Neurodevelopmental Disorders

#### Rangon, C.M.

Neurodevelopmental disorders, including autism spectrum disorders, are complex and heterogeneous disorders. These disorders affect a large number of children, and represent a tremendous human, emotional, social and economic burden. The progressive decrypting of the pathophysiology of psychiatric disorders has led to the blurring of the frontiers between child neurology, pediatric psychiatry, developmental neurobiology, cognitive neurosciences, and social sciences.

Nevertheless, currently available treatments are still limited. Auriculotherapy might represent an efficient alternative because of its action on brain plasticity without side effects.

Young patients that could benefit from auricular treatments are presented. A special emphasis is laid upon the therapeutic strategy used and the observed outcome. 

Randomized, double-blind, multicentric clinical trials are urgently needed and should take advantage of this 2017 Singapore Symposium.

### 2.5. Auricular Acupuncture Research in the People's Republic of China During the Past 10 Years

#### Zhao, B.X.

Background: Since the end of 1950s, auricular therapy and clinical application have attracted much attention from a large number of researchers home and abroad, which greatly stimulated the enthusiasm of auricular acupuncture among the whole Chinese acupuncture societies in china. On the basis of sorting out the ancient literature, the Chinese researchers have carried out countless clinical researches and exploration. In order to analyze diagnosis and treatment with auricular points during the last ten years, from 2007 to 2016 in China, China—Tsinghua Tongfang “CKNI” (China National Knowledge Infrastructure) Journal Full-text Database was applied for data retrieval source article about the application of auricular points, and main research program results are summarized in order to provide a theoretical basis for understanding and development of auricular therapy in China and all over the world.

Methods: To analyze the diagnosis and treatment of auricular points during the past ten years (2007 to 2016) in China, China—Tsinghua Tongfang “CKNI (China National Knowledge Infrastructure) Journal Full-text Database” was applied to search the relevant research articles with the “auricular acupuncture” as the key word, which are about the history, the overall situation, standardization and so on. 

Results: According to data mining on the recent ten years’ literatures, high-frequency symptoms treated by auricular acupuncture, high-frequency diseases treated by auricular acupuncture, qualities of different levels of researches, the overall research progress on auricular acupuncture were summarized, and existing issues were found and development prospects are analyzed.

Conclusions: Auricular acupuncture for the treatment of various diseases has been widely used in China, and gain very good clinical effects for some specific diseases. However, the auricular acupuncture as a kind of treatment in micro needle system still has a long way to go. The further study of auricular acupuncture should be more comprehensive and in-depth, also the quality of scientific researches at all levels have to be further improved.

### 2.6. Standard Definition of Auriculotherapy and Ear Acupuncture- (Group Discussion)

#### Nogier, R.

*This is an open discussion and will be chaired by Prof. Gerhard Litscher (Austria) and Dr. Im Quah-Smith (Australia)*.

Dr. Nogier presents defintitions of auriculotherapy according to GLEM and compares it with ear acupuncture.

### 2.7. The New International Nomenclature for Earpoints: The Development of the 2D Coordinate Ear

#### Bahr, F.; Wojak, W.

*This important discussion will be chaired by Prof. Gerhard Litscher (Austria) and Dr. Im Quah-Smith (Australia)*.

The national and international comparability of different ear localisations would have been desirable since the first publication by Nogier in 1957, because different researchers, sometimes from very distant locations, published their results on ear localisations, which could be compared hardly or not at all. The reason was that they were talking about different “standard ears”, although it is clear that there can be no such thing as a standard ear, but nevertheless there is the need to achieve comparability, which is why one has to make compromises with regard to abstraction. It may help to consider that not even the left and right ear of a person are identical. As far as we know, Paul Nogier introduced a standard ear with coordinates arranged from A to O and A to Z, respectively, for the first time in his book “De l’Auriculothérapie àl’Auriculomédicine” in 1981. He subclassified certain areas using colours and markings because he did not deem the grid boxes sufficient for the description of certain important reflex points. 

In our seminars we quickly found that this coordinate ear not only enabled us to achieve the desired international comparability, teaching was also made a lot easier. In workshops the ear-coordinates have always been well received by the participants, who are finally equipped with clear instructions. In the meantime, the coordinate ear has been in use in several European countries (Figure 3), and it has stood the test in daily practice. We have begun to define nearly all important ear acupuncture points with regard to coordinates. By and by we will classify the well-known ear points over the next months and publish them in our journal. Furthermore, we will also apply the coordinate system to the back of the ear and publish the point descriptions.

### 2.8. Potential for the Combination of Auricular Point Electrotherapy and TES, the Significance and Prospects

#### Cheng, K.

Background: Auricular point electrotherapy is found to be about to regulate corresponding functions of the cerebral cortex, for the electrical stimulation of specific auricular points can produce specific neurophysiological responses in different regions of the brain. Transcranial electrical stimulation (TES) is widely applied in brain function research and treatment. This report is going to discuss the possibilities and impact of the combination of the two technologies.

Methods: Relevant literature of the past ten years (2007–2017) is comprehensively, systematically searched and collected, processed by modern statistics after preliminary analysis. The results are analyzed and discusses.

Results: Auricular point electrotherapy and TES are both widely applied in clinical research. The mechanisms and target points through with they function are different. Stimulation induced by TES is not concentrated enough, whereas auricular points are smaller and the targeting is stronger.

Conclusions: Auricular point electrotherapy and TES could form a comprehensive approach in clinical application as auricular transcranial electrical stimulation (ETES). It has considerable prospects in future clinical practice.

### 2.9. Neuropathic Pain: Auriculotherapy Potential

#### Cornelia, D.M.; Angelo, S.

Auriculotherapy is apt to treat pathological conditions based on a neurophysiological pattern. It acts on the neural networks, eliciting action potentials, urging and rebalancing hormone and neurotransmitters secretion. The fastest and the best results are obtained in treating painful syndromes of whatever origin.

It has been acquired acupuncture to elicit synthesis of endorphin, enkephalin, dynorphin, serotonergic, noradrenergic, and non-opiod neurotrasmitters. 

It is conceivable the central-mediated effects of acupuncture to be involved in suppression of neuropathic pain, too [3–5]. 

We aim to do an excursus of approaches to different patterns of neuropathic pain, starting with the Marco Romoli’ s intellectual contribution and experience in patient observation and auricular diagnosis, without neglecting other methods, conceived on the pathophysiological basis for neuropathic painful syndromes [1,2,6,7].

Some cases, mostly unpublished, treated by scientific western auriculotherapy, are reported. 

Nevertheless, it is necessary to bring efficacy trials, by means of applied research on suitable sample size of patients. To do this, the biggest challenge is to find financial and human resources, but the main requirement for researchers and clinicians, around the world, is to improve the communication skills to exchange details about their work and share the results of their ongoing studies.

1. Romoli, M. *Agopuntura Auricolare*; UTET: Torino, Italy, 2003.
2. Woolf, C.J.; Mannion, R.J. Neuropathic pain: Aetiology, symptoms, mechanisms and management. *Lancet* **1999**, *353*, 1959–1964.
3. Franconi, G.; Manni, L.; Schroeder, S.; Marchetti, P.; Robinson, N. A Systematic Review of Experimental and Clinical Acupuncture in Chemiotherapy-Induced Peripheral Neuropathy. *Evid. Based Complement. Altern. Med.* **2013**, *2013*, 516916.
4. Jung, W.S.; Chen, L. Acupuncture and Neuropathic Pain Management. *Med. Acupunct.* **2013**, *25*, 261–268.
5. Rabischong, P.; Terral, C. Scientific Basis of Auriculotherapy: State of the Art. *Med. Acupunct.* **2014**, *26*, 84–96.
6. Alimi, D. Xerostomia induced by radiotherapy. *Ther. Clin. Risk Manag*. **2015**, *11*, 1149–1152.
7. Stanton, G.; Rangon, C.-M. The Scientific Auriculotherapy Diploma Program of the Universities of Paris XI and XIII. *Med. Acupunct.* **2014**, *26*, 118–124.

### 2.10. Laser Acupuncture for Ear and Body Points in Arterial Fibrillation

#### Weber, M.

Atrial fibrillation (AF) is an abnormal heart rhythm characterized by rapid and irregular beating of the heart. In many patients it starts as brief periods of abnormal beating which become longer and possibly constant over time. Many episodes have no clear symptoms. Some patients complain of heart palpitations, fainting, light headedness, shortness of breath, or even chest pain. On the long run the disease is associated with an increased risk of heart failure, dementia, and stroke. 

AF is a type of supraventricular tachycardia. AF is linked to several forms of cardiovascular disease, but may occur in otherwise normal hearts.

Atrial fibrillation is clearly diagnosed by electrocardiogram (ECG). Characteristic findings are the absence of P waves, with disorganized electrical activity in their place, and irregular R-R intervals due to irregular conduction of impulses to the ventricles.

The main goals of treatment are to prevent circulatory instability and stroke. Rate or rhythm control are used to achieve the former, whereas anticoagulation is used to decrease the risk of the latter. If severe cardiovascular impairment occurs due to uncontrolled tachycardia, immediate cardioversion is usually performed.

The Chinese Medicine (CM) treatment of arrhythmia (palpitations) generally involves arriving at the appropriate TCM diagnosis or pattern. This pattern within the individual is what treatment is based on not the general condition.

The following patterns may represent the underlying contributing factors for the development of arrhythmia (palpitations): Heart Qi Deficiency, Heart Yang Deficiency, Heart Yin Deficiency, Heart Yin Deficiency with secondary Heart Fire, Spleen Qi Deficiency, Spleen Yang Deficiency, Kidney Yang Deficiency, Kidney Yin Deficiency, Liver Qi Stagnation, Blood Stagnation, Blood Heat.

It is in example important to carefully distinguish Heart Fire from Empty Heat in the Heart, because they require absolutely different treatments: the wrong treatment could, theoretically, make the patient worse.

From 2014 to 2016 we treated 12 patients with AF (9 men and 3 female). All of them presented with heat in the blood and at least some extend of heart fire. In all patients electro cardioversion had been performed with only temporary success. One patient relapsed less than one hour after cardioversion, the longest persisting period of normal heart rhythm after cardioversion was 10 days.

In all patients we treated: Neiguan (Pericardium 6), Shenmen (Heart 7), Shaofu (Heart 8), the front Mu-Point Jiuwei (Renmai 15), Sanyinjiao (Spleen 6), Taiyuan (Lung 9), Xingyian (Liver 2). The treated auricularpoints treated were: shenmen, heart and the Bahr points Valium and Haldol. 

All patients were treated by laseracupuncture (RJ—Laserpen max. 500mW; 810 nm/infrared). The local energy doses was given at 4.0 J/body acupuncture point and 0.5 J/ear acupuncture point. 

In the literature there are a few publications suggesting that acupuncture at traditional Chinese acupuncture points may be effective in the treatment of AF [1]. Lombardi and co-workers observed that acupuncture of the Neiguan (Pericardium 6) spot was associated with an antiarrhythmic effect, which was evident in patients with both persistent and paroxysmal AF.

In addition after the CM treatment the patients were radiated by LowLevelLaserLight (LLLT) with cluster probe (RJ—Physiolaser Olympic; Cluster Probe 516C superpulsed (directed beam) 5 × 904 nm/30 Watt) positioned under sternum directed towards heart (mimic 4-chamber view).

LLLT is targeting mitochondria. All human cells with the exception of adult red blood cells contain mitochondria and these are specific targets for laserlight. The more active a human cell is, the higher is the number of mitochondria.

In heart muscle cells, mitochondria take up 36% of the volume. Most human organs contain 500 to 2000 mitochondria per cell [2]. The double membranes of the mitochondria consist of proteins and fats. Especially the protein structures (flavins, cytochromes, and porphyrins) have a pronounced ability to absorb laser beams [3]. The absorption boosts the activity of the enzyme, such as flavin dehydrogenase and cytochrome oxidase [4]. T. I. Karu stated as early as 1988 that the antenna pigments of the respiratory chain absorb low-level laser light directly, thus boosting ATP production [5].

Finally in all patients’ blood work was performed: whole blood count, thyroid hormones, omega-3-index, homocysteine, CoEnzyme Q10, vitamin D3, L-carnitin magnesium, copper and potassium (in whole blood). In cases of deficiency the patient were initially treated on therapeutic level, maintenance was given at [6]: omega-3-fetty acids 3 g/day; coenzyme Q10 at 100–300 mg/day, vitamin D3 2000 IE/day, L-carnitin 2000 mg/day, magnesium 600 mg/day, copper 1 mg/day.

In all patients we were able to cease AF and return to normal sinus rhythm during treatment or within 6 h. 8 of 12 patients had relapse of AF within two weeks, however, responded immediately at follow up appointments. Only in one of the 12 patients radiodiofrequency ablation of pulmonary vein was performed. 

These preliminary data, observed in a small observationsgroup of AF patients, need to be validated in a larger population with control group. However, we strongly suggest that Laseracupuncture targeting mitochondria may be an effective non-invasive and safe antiarrhythmic tool in the management of these patients.

1. Lombardi, F.; Belletti, S.; Battezzati, P.M.; Lomuscio, A. Acupuncture for paroxysmal and persistent atrial fibrillation: An effective non pharmacological tool? *World J. Cardiol.* **2012**, *4*, 60–65.
2. Gvozdjáková, A. *Mitochondrial Medicine. Mitochondrial Metabolism, Diseases, Diagnosis and Therapy*; Springer: Berlin, Germany, 2008; p. 1.
3. Warnke, U. *Ein Elementarer Halbleiter-Laser-Wirkmechanismus bei Katalytischen Prozessen und Redoxvorgängen*; Monduzzi, Ed.; Congress on Laser Int.: Pologna, 1985; p. 225.
4. Tiphlova, O.A.; Karu, T.I. Role of primary photoacceptors in low-power laser effects: Action of He-Ne Laser radiation on bacteriophage T4-Escherchia coli interaction. *Laser Surg. Med*. **1989**, *9*, 67–69.
5. Karu, T.I. Molecular mechanism of the therapeutic effect of low-intensity laser irradiation. *Lasers Life Sci.* **1988**, *2*, 53–74.
6. Gröber, U. *Mikronährstoffe; Metabolic Tuning, Prävention, Therapie*, 3rd ed.; wbg Stuttgart 2011; pp. 453–454.

### 2.11. The Effects of Auricular Electro-Acupuncture on Ameliorating the Dysfunction of Interstitial Cells of Cajal Networks & nNOSmRNA Expression in the Antrum of STZ-Induced Diabetic Rats

#### Zhang, Z.H.; Chen, H.; Zhu, W.J.; Lu, J.; Fan, J.J.; Sun,L.N.; Feng, X.K.; Liu, H.; Wang, Y.Q.

Background: Interstitial cells of Cajal (ICCs) and nNOS play a crucial role in diabetic gastrointestinal dysmotality (DGD). Our previous study found that electro-acupuncture (EA) on ear point ‘stomach’ could repair the gastric dysrhythmias in rats induced by rectal distention (RD) after meal. However, little was known about the possible effect of auricular electro-acupuncture (AEA) on diabetic rats. Thus, we designed this study to investigate the effect of AEA on streptozotocin(STZ)-induced diabetic rats.

Method: Male Sprague-Dawley (SD) rats were injected with STZ, at the end of the 8th week after injection fourty diabetic rats were randomly divided into four groups and received 2 weeks treatment (10 times) respectively: control group (CON, *n* = 10, no stimulation), sham auricular electro-acupuncture group (SEA, *n* = 10, low frequency EA on earlobes), auricular electro-acupuncture group (AEA, *n* = 10, low frequency EA on ear point ‘stomach’), and ST-36 groups (ST-36, n + 10, low frequency EA on ST-36). Gastrointestinal (GI) motility was measured by GI transit rate. ICCs (c-kit + expression) in antrum were analyzed by immunohistochemistry and western blotting. NO level in blood serum were detected by Griess Reagent, and nNOSmRNA expression in antrum were determined by Real-time PCR.

Results: GI transit rate and ICCs(c-kit+ expression) in antrum of AEA group have the tendency to increase compared with CON group, but had no statistics difference (*p* > 0.05). nNOSmRNA expression in antrum of AEA group was dramatically increased compared with CON group (*p* + 0.037).

Conclusion: Low frequency EA on ear ‘stomach’ point could ameliorate the ICCs networks partly and up-regulate nNOS mRNA expression significantly in gastric antrum of STZ-induced diabetic rats, which may have benefits on regulating the GI motality.

### 2.12. Cryotherapy as a Modality in Auriculotherapy

#### Alimi, D.

Background: To diversify the range of therapeutic instruments in Auriculotherapy.

Methods: fMRI was performed in order to test the efficiency of cryonic needles. Multicentric Study comparing Semi-Permanent Needles and “Cryonic Needles” during 16 months, involving 4500 patients; suffering of different chronic and acute diseases. 

Results: A gain is noted in all the pathologies as well naïve patients of Auriculotherapy (72% of positive responses for cryonic needles, versus 70% of positives responses for semi permanent needles), as patients knowing Auriculotherapy by semi permanent needles (63% of positive responses for cryonic needles, versus 60% of positive responses for semi permanent needles). Conclusions: These “cryogenic needles” allow an auriculotherapy without bleeding, almost painless, risk-free, eliminating all of the inconveniences of mechanical needles, while at the same time maintaining their efficacy.

## 3. Lectures 

### 3.1. Four-Arm Placebo Controlled Study Using Auriculotherapy for Elderly with Osteoarthritic Knee

#### Suen, L.

Background: Osteoarthritic (OA) knee is a common condition in the elderly. Patients may develop severe pain and impaired physical functions as the disease progresses. Nonsteroidal anti-inflammatory drugs mainly focus on musculoskeletal pain relief but lead to side effects, such as gastrointestinal hemorrhage. As such, non-invasive complementary techniques with minimal side effects on OA knee should be explored. Auriculotherapy (AT) is a therapeutic method where specific points on the auricle are stimulated to treat various disorders. The therapeutic effect of AT may be optimized by applying magneto-auriculotherapy (MAT) after laser auriculotherapy (LAT). It is hypothesized that MAT offers continuous stimulation of acupoints after laser treatment as long as the magnet pellets on the ears are in situ.

Materials and Methods: In this four-arm, placebo-controlled trial, both the participants and the assessor were blinded to the grouping allocation. Participants (aged ≥ 60 years; *n* = 66) with the defined OA knee condition were recruited and randomly divided into four groups: Group 1, treated with combined MAT and LAT (i.e., LMAT; *n* = 17); Group 2, treated with deactivated LAT followed by MAT (*n* = 16); Group 3, treated with LAT and placebo MAT (*n* = 19); and Group 4, treated with placebo MAT and placebo LAT (*n* = 14). The treatment was delivered to six specific auricular points, namely, shenmen, knee, spleen, liver, kidney, and subcortex, three times a week for 6 weeks. The participants were assessed using a numerical rating scale of pain (NRS), timed-up-and-go test (TUGT), and standard goniometer measurements to measure the active and passive ranges of movement of the knees during flexion and extension. Outcome measures were conducted at baseline, end of 6-week treatment, and at 6- and 12-week follow-up periods.

Results. The demographic and clinical characteristics of the participants were not significantly different among the four groups at baseline. Preliminary analysis results indicated that NRS, TUGT, and active/passive knee flexion and extension were not significantly different at baseline and after the therapy among the four groups. However, within-group comparison showed that majority of the subjective (NRS, TUGT) and objective parameters (knee flexion) significantly differed before and after the intervention in subjects who received LMAT, MAT, and/or LAT (i.e., groups 1–3). The placebo group showed the least relative difference in the parameters. In addition, there was notably improvement in a number of symptoms in the LMAT group, such as sleep condition and decreased nocturia. The overall satisfaction toward AT was high (8.14 of 10). Moreover, the majority of the participants (*n* = 56, 85%) indicated that they will recommend AT to the others.

Conclusion: Subjects who received LMAT, MAT or LAT alone presented improved treatment outcomes than those who were given the placebo treatment. Future studies should employ a larger sample size to identify the most suitable treatment protocol for AT in managing OA knee(s) in the elderly.

### 3.2. Auriculotherapy in Sleep Medicine

#### Stanton, G.

This 30 min lecture will briefly review the clinical significance of sleep disorders, the anatomy and physiology of sleep, and their somatotopic correspondence to auriculotherapy diagnosis and treatment strategies. Examples of clinical applications will focus primarily on the auriculotherapy of insomnia. 

### 3.3. Clinical Efficacy Evaluation on Pain and Motor Dysfunction of Ischemic Stroke Patients with Phase I Shoulder-Hand-Syndrome (SHS) Treated by Auricular Acupuncture Therapy

#### Meng, X.N.

Background: Shoulder-Hand-Syndrome (SHS) is a common complication of stroke patients, which has already become the third most-common complications of stroke, with clinical high incidence, severe prognosis with disability, leading to the serious influence to the individual patient, family and society. The pathogenesis is complex, and the expense burden is heavy. Traditional Chinese medicine treatment for SHS is with the curative effect, less adverse reactions, low cost, with good prospects for development. Auricular acupuncture, as one kind of micro-system acupuncture, with the better clinical analgesic effect, now is simple and easy to operate, with less pain, less cost, high patient acceptance, and suitable for clinical application

Methods: More than 76 cases of post-stroke shoulder hand syndrome have been collected for this research, and they have been divided into two treatment groups. The standard acupuncture treatment group will be given body acupuncture and rehabilitation, whereas the other treatment group will receive auricular acupuncture as well as body acupuncture and rehabilitation. The results of this research are not yet finalized as the study is ongoing, but preliminary findings will be ready by the time of the symposium in August, 2017. The statistical effectiveness of auricular acupuncture in combination with the other Chinese medicine will be measured by a pain rating on a Visual Analog Scale (VAS), the Fugl-Meyer upper limb motor function score and an evaluation index of shoulder joint mobility.

Results: The research is in process. So the final findings for this study will be presented at the seminar in Singapore.

Conclusions: The final findings for this study will be presented at the seminar in Singapore.

### 3.4. Acupuncture as an Adjunctive Treatment for Addictive Disorders

#### Chan, H.; Chin, X.Y.; Guo, S.

Background: Addiction is a complex condition, a brain disease that is manifested by compulsive substance use or behaviour despite harmful consequence, with accordance to the Diagnostic and Statistical Manual of Mental Disorders, Fifth Edition. For most healthcare institutions, a combination of medication and individual or group therapy is offered to help-seeking individuals with addiction disorders. Established in 2013, the Acupuncture Clinic for addiction management aims to offer a drug-free adjunctive treatment with low side-effects, which may increase the individuals’ treatment engagement and their overall health.

Methods: A combination of auricular and body acupuncture was performed on all patients. Various tools of treatment outcome monitoring were utilised, namely Addiction Severity Index—Lite and the self-reported craving level to understand the symptom severity of addiction; Personal Well-being Index to measure the quality of life, and the lifestyle questionnaire to have a more comprehensive understanding. Repeated measures were administered to understand the progression of the patients.

Results: Preliminary findings showed significant improvements in the symptoms severity and overall well-being satisfaction. It was also found that the various aspects of the individuals’ lifestyle had improved during the course of acupuncture treatment. Comparison with the Treatment As Usual (TAU) group showed that Acupuncture in combination with TAU has a positive effect on treatment engagement, in which there were significantly more repeat doctor’s and allied health visits than those who only received TAU.

Conclusions: Further studies could be carried out to provide a clearer picture on the acupuncture treatment’s effectiveness and patients’ treatment engagement.

### 3.5. Ear Seeds for Urgent Care in Western Clinic

#### Alabaster, J.

Background: Auricular acupucture using vaccaria ear seeds for musculoskeletal conditions, including back pain, neck pain, shoulder pain, migraines and anxiety in an acute care western medical clinic.

Methods: points used according to nogier, contralateral ear used to treat affected side.points used shenmen, kidney, adrenal, occiput, and anatomical points as needed. Pain scale rating 1–10 pre and post treatment.

Results: 50–80% of patients reported immediate improvement of symptoms ranging from 50–90% improvement.

Conclusions: In the worst case scenario there was no relief of symtoms but no adverse effects. In the best outcomes a significant reduction in symptoms was achieved. Overall patient satisfaction was high.

### 3.6. Treatment of Acne by Auricular Points Bloodletting:A Randomized Controlled Study of Small Samples

#### She, Y.F.

Acne is a chronic inflammatory disease of the sebaceous gland, more common in the face, In TCM the incidence of acne is due to the “lung” “wind” and “heat” and “depression” “heat” “blood”. The ear is closely related to the viscera and meridians, auricular points can adjust the function of organs and meridians. Bloodletting therapy has a good effect of clearing heat and detoxification. Based on the above theory, we speculate: Auricular point pricking technology has the potential for the treatment of acne. 

Methods: Sixty patients with acne vulgaris were randomly assigned to two group (experimental group and control group) in 1:1 scale. The patients in the experimental group were treated with auricular points bloodletting combined with auricular points acupressure .The control group was treated with pure auricular points acupressure. The patients in the two groups were treated 3 cycles. Before and after each treatment cycle, recorded index as acne area, quantity, type, color, pain, itching etc. Integral of damaged skin were the total score of each index, Curative effect index was calculated according to integral of damaged skin .After three treatment cycles to observe the change of acne severity classification and the clinical curative effect.

Results: After 3 cycles of treatment in 2 group: the total effective rate of the experimental group was 80%, the control group was 50%; the severity of acne in the experimental group was significantly lower than that in the control group (*p* < 0.05); no adverse reactions were observed in all the patients.

Conclusions: Auricular point pricking combined with the auricular points acupressure method in the treatment of acne curative effect was more significant than auricular points acupressure method. This method which good safety.

Registration and Funding: Hebei province science and technology support projects (132777263).

### 3.7. Operation Specification Selection of Auricular Therapy with Chinese Characteristics

#### Liu, J.H.

Background: The hospital of Traditional Chinese Medicine in Foshan to undertake the project in 2014. The Project is “The technical operation specification of preventive treatment of disease of TCM: Auricular Diagnosis and Treatment”, which is supported by the state administration of traditional Chinese medicine.

Methods: After nearly 2 years of research and practice, finishing 286 cases of similarity test by 12 cooperation units in 2016, in the mid-term review, the proposal of standards was passed by the China association of Chinese medicine.

Results: The clinical conformance testing was completed in the efforts of cooperate whole heartedly whit dozen peer cooperation unit in March 2016. It will be issued in the near future if the proposal be passed the review by the national instruction group of the technical operation specification of preventive treatment of disease of TCM and China association of Chinese medicine. This is the operation specification selection of auricular therapy with Chinese characteristics.

### 3.8. Auriculotherapy in Vitiligo Treatment

#### Kuzulugil, A.; Practitioner, P.

In this oral presentation I would like to present a Vitiligo patient who has been treated by Auriculotherapy. A 54 year-old female patient admitted to my clinic with complains of depigmented lesions appear on 3 different areas of her face which appeared 3 months prior. At first a dermatologist examined her. Diagnosis was vitiligo and topical treatment of vodoid lipocream was recommended. After a three months treatment period the patient quit the treatment as no benefit was gained. The patient agreed to start Auriculotherapy after admission to the clinic. Ear acupuncture was applied bi-weekly. The points were determined by way of VAS and were verified with Agiscop DT. These points were consistent with the pathophysiology of the disease. ASP classic semi-permanent needles were used throughout the treatment. The total area of depigmentation was decreased gradually during the treatment period. The improvement in lesions started from periphery to the centre of the depigmentated area. On the 22nd week of the treatment, the skin was completely normal. In conclusion it can be said that Auriculotherapy has a regulatory role in the function of pigment cells.

### 3.9. Painless Laser Acupuncture (PLA) for Quit Smoking

#### Lim, R.

Introduction: With the advances in the field of medical Sciences today, smoking among the young has become a “epidemic” that all Health Authorities worldwide are finding it hard to curb. Most smokers wanted to quit smoking but the dependency and cravings of Nicotine had putted them off. This report was a collation of experience from more than a hundred of patients who seek treatment at the Laser Acupuncture Centre, Singapore since 2013. The approach to tackle this recalcitrant challenges was based on the holistic intervention by applying the Traditional Chinese Medicine Principles in Acupuncture. Although Acupuncture is common practice in China, Europe, USA and many parts of the world, it is still not popular due to needle phobia. Painless laser acupuncture (PLA) set out to overcome this fear by using the Laser Light modality which provides great comfort to the patients.

Materials and Methods: The ‘RJ’ Physiolaser Olympic system was used with Two probes: Probe A—638 nm/150 mW which delivered 45 Joules/cm^2^ at 5 min. Each auricular point received 3.5 Joules. Probe B—810 nm/300 mW which delivered 180 Joules/cm^2^ at 10 min. Each body point received 7.5 Joules. Total Energy dosages delivered per session was 225 Joules/cm^2^. Combination of Auriculotherapy and Body Meridians points were used. Depending whether the client was right or left handed, the opposite Ear was used. The Auriculotherapy Points selected were Shenmen, Heart, Lung, Endocrine, Brain, Adrenal, Stomach, Liver, Kidney and Finger. The Body meridians used are Lung, Pericardium, Heart, Kidney, Large Intestine, Stomach, Spleen, Ren and Du. Each client received 7 sessions with first 3 sessions on daily basis. The first week 4 sessions and second week was 2 sessions and third week one session.

Results: Most patients were executives in their early 40s and 50s who are highly motivated and stressed due to the nature of their jobs. The reason for seeking treatment was also partly due the strain of their health and support from their family to quit smoking. Generally, most smoke less than one (1) pack of 20 cigarettes. In general, patients did not feel ‘difference’ after the first session. However, should they smoke the 1st cigarette post therapy, the feedback was that they no longer enjoyed its taste as before. By the 2nd session, they said the cravings had reduced. By the time after the 3rd session most reported that they no longer finished the stick of cigarette they smoke as the taste was awful. Many did not smoked at all by the 4th sessions, more than 80% people feedback that they had no urge to smoke. For those who are serious and genuine to quit smoking stopped after completing 4 to 5 sessions. 

Discussion: Smoker had higher neurotransmitters like endorphin, dopamine and serotonin in the blood than a non-smoker. Therefore, when a smoker stop smoking, these neurotransmitters will be reduced thus withdrawal set in. The Painless Laser impulses applied in Auriculotherapy will trigger the Brain to stimulate the release of neurotransmitters naturally and the Auricular points were nearest to the Brain, therefore the effect to activate the Autonomous system to change its Bio-chemistry effectively. Also, the Body points improved their quality of sleep, calmness, energy and enjoyed the taste of food. By the Law of Physical science, the Laser light energy has unique properties with its Magnetic (YIN) components perpendicular to Electric (YANG) components. This balanced energy with Yin-Yang characteristic fulfilled the principle of Classical Acupuncture without the use of “Needle and Moxa roll” modalities. The balanced properties of Laser energy and the dosage of each point could be quantified increased treatment efficacy. Nevertheless, the bottom line regarding quit smoking was the individual intention to kick the bad habits. Once they had that focus, the chances of success could always be assured with treatment. 

Conclusion: Painless Laser Acupuncture in Auriculotherapy provided a great platform for all smokers to give up smoking easily! We strongly encourage all practitioners to work towards this goal with more clinical to help smokers. We call on Health ministry worldwide to support research on Auriculotherapy to tackle this challenge.

### 3.10. Auricular Chromotherapy in the Treatment of Psychological Trauma

#### Yoshizumi, A.; Asis, D.; Luz, F. 

Auricular Chromotherapy has shown promising results in the treatment of psychological trauma. With its relatively easy and quick technical application and the good results produced, this procedure may be an indispensable tool for physicians. However, its mechanism of action is not yet completely understood. This work mentions: (i) the steps followed before the first application in 30 patients in Santa Fé (Argentina) where the technique was created [1]; (ii) the results of 160 cases (134 women, 26 men, aged 20 to 60) seen in São Paulo (Brazil) with a 93% success rate; (iii) some possible lines of research for the future.

1. Asis, D. *Cromo-Psicoterapias—Cromoterapia Auricular, Cromo-TIC, Anteojos Cromoterapeuticos*; Ernesto Julián Friedenthal: Buenos Aires, Argentina, 2009.

### 3.11. Magnetic Non-Invasive Acupuncture for Infant Comfort (MAGNIFIC) in Heel Pricks—A Randomised Controlled Pilot Study

#### Chen, K.L.; Lindrea, K.B.; Quah-Smith, I.; Schmölzer, G.M.; Daly, M.; Schindler, T.; Oei, J.L.

Background: Both pain and common pharmacological analgesics may adversely affect newborn neurodevelopmental outcomes. We investigated the feasibility and safety of magnetic acupuncture (MA) as an adjunctive analgesic for heel pricks in newborn infants. 

Method: After parental consent, infants requiring heel pricks for blood collection were randomised to either MA (*n* = 21) or placebo (P) (*n* = 19) between 15th August–10th November 2016. Five MA or placebo stickers were placed on auricular acupuncture sites on each ear for 3 days by an unblinded investigator. Pain responses were assessed with the Premature Infant Pain Profile (PIPP) by blinded clinicians 15 min before and after and during each heel prick. The study was registered in the Australian and New Zealand Clinical Trial Registry (ACTRN12616001229460).

Results: Infants were similar in birth weight (MA: 2303 g, P: 2104 g) and age at heel prick (MA: 5.3, P: 4.5 days). Mean (SD) PIPP scores were similar before (MA: 1.7(1.4), P: 2.1(1.9)) and after (MA: 1.6(1.4), P: 2.1(1.7)) heel pricks but were lower in MA infants during heel pricks (MA: 5.9(3.7) v P: 8.3(4.7), *p* = 0.04). One-way ANCOVA modelling demonstrated a significant effect of MA on heel prick PIPP scores even after controlling for analgesia (*p* = 0.043, eta^2^ = 0.07). No differences were noted in heart rate, SpO_2_ or incidence of adverse effects (e.g., local skin reactions, sticker displacement).

Conclusions: Auricular MA is feasible in neonates and may reduce PIPP scores during common procedures like heel pricks. Further studies are required to determine the impact of MA on other painful procedures and on neurodevelopmental outcomes.

### 3.12. The Hidden Information in Your Brain Concerning Your Future: How to Influence Your Darma and Your Karma

#### Bahr, F.

This very special Saturday afternoon workshop (with introductory talk on Friday morning) helps the clinician correlate the auricular access to find weak points in the constitution, which might be harmful for the future of the patient.

With semipermanent needles or the new needle-implants (soluble within 3 to 6 weeks) we treat the earpoint, which has been detected with auriculomedicine technique (Nogierpulse).

### 3.13. The Treasure House of Auricular Diagnosis And Therapeutics

#### Aung, S.

Auricular Diagnosis is one of the most important diagnostic methods in Traditional Chinese Medicine (TCM). Since the ear is considered to be a microsystem of the human body due to its anatomical association with the inverted human embryo, medical diagnosis may be obtained through the ear by examining the physical features. Signs and symptoms such as colour changes, formation of nodules, visible appearance of blood vessels, moles, and auricular lines are vital in the procurement of the diagnosis of a patient, capable of diagnosing problems with 60–80% accuracy. The anatomical connection between the ear and parts of the body goes both ways; pathological problems elsewhere in the body can not only be diagnosed via the ear, but also treated. Pathological points are detected by an electrical detector, but can also be located by the *auriculocardio reflex* (first described by Paul Nogier), the sensitivity and pain reaction to applied pressure. Giving acupuncture to the corresponding ear part will treat the problem area in the body. The advantage of treating via the ear is convenience and easy accessibility—therefore, easy adjustment of needles and placement of ear patches. Ear patches can mediate pain, anxiety, tension, and provides constant stimulation to the ear. Hence, auricular diagnosis is vital in Traditional Chinese Medicine for its reliability in diagnosis and its therapeutic value in providing treatment, particularly for addictions or behavioural problems and for follow-ups.

### 3.14. Active Somatic and Psychic Ear Acupuncture Points in Newborn Infants with Neonatal Abstinence Syndrome (NAS)

#### Raith, W.; Kurath-Koller, S.; Pansy, J.; Mileder, L.; Schmölzer, G.; Urlesberger, B.

Background: Neonatal Abstinence Syndrome (NAS) occurs within the first days after birth in newborns of mothers with a history of drug abuse or mothers undergoing replacement therapy. To determine the presence of active ear acupuncture points in newborn infants with NAS was the aim of the study.

Methods: Between 3/2009 and 11/2014 newborn infants with maternal history of drug abuse or maternal replacement therapy admitted to Neonatal Intensive Care Unit at the University Hospital Graz were included. Active ear acupuncture points were identified using an “acupuncture-point detector” (PS 3© Silberbauer, Vienna, Austria). An integrated optical and acoustical signal detects the ear points, which were then assigned to the ear map. 

Results: A total of 31 newborn infants were eligible; one infant was excluded because the mother had already weaned herself off opiates prior to admission. The excluded infant did not develop signs of NAS with a Finnegan Score of three points and no detectable active psychic ear acupuncture points. 

In all included newborn infants with NAS, active ear acupuncture points were identified: In 100% of infants we identified the psychovegetative rim as the most common active somatic area, which was followed by a few somatic and psychic ear acupuncture points. Furthermore, in all infants with symptoms of NAS we identified active psychic ear points, of which the most frequently found points are Frustration–point and R-point.

Conclusion: In all included infants with NAS we identified active somatic and psychic ear acupuncture points. 

### 3.15. Pulsed Magnetic Fields on the Auricular Zones: A Preliminary Study for the Choice of Pulsed Frequencies through Auricular Medicine

#### Vulliez, C.; Becu, P.

Background: Pulsed magnetic field (PMF) is an important non- invasive alternative therapeutic option that has been investigated in several pre-clinical and clinical studies. For example, transcranial magnetic stimulation is increasingly used as a treatment for neurological dysfunction, and extremely low frequency pulsed magnetic fields have been shown to induce Faraday currents and measurable effects on biological systems. However, there are few if any studies about the effects induced by different frequencies.

Methods: In this research project, we applied the principle of transcranial pulsed magnetic field stimulation directly to the whole ear bilaterally, with subjects wearing a helmet equipped with transcranial electromagnetic auricular applicators.

First we carried out a double-blinded diagnostic trial involving 100 patients, applying five different PMF stimulations while monitoring the VAS (the Nogier signal). The five different PMF simulations selected had previously been applied to a small group of 17 subjects whose pathologies appeared to respond to them using the VAS as a detection method.

Second, we carried out a diagnosis and treatment protocol on 260 patients presenting such pathologies, using those frequencies in 3 different programs on both ears with a prototype, and followed them up during 3 to 8 months. During that time, we noted improvements from 0 to 3+, along with other relevant information. 

Results: All patients followed in the diagnosis and treatment arm showed anywhere from a notable to excellent improvement, progressive over time, but present following the first session.

Conclusion: Pulsed magnetic fields with appropriate frequencies applied to the whole ear bilaterally showed evidence for therapeutic efficiency.

### 3.16. Active Ear Acupuncture Points in Sick and Healthy Term and Late-Preterm Neonates: A Blinded, Controlled, Observational Trial

#### Stadler, J.; Flucher, C.; Kurath-Koller, S.; Tritschler, N.; Urlesberger, B.; Raith, W. 

Background: In Europe, the French physician Paul Nogier was able to demonstrate that all body organs are represented as reflex zones on the outer ear, so called ear acupuncture points. Active points can be present and detectable during disease and therefore, can be used in diagnostic and therapeutic concepts. However, there is only little known about active ear acupuncture points in neonates. Therefore, this blinded, observational trial was conducted to locate active ear acupuncture points in healthy and sick neonates and to investigate the influence of birth mode on number of active ear points.

Methods: Ear acupuncture points were detected on both ears by an electrical point search device (PS3© by Silberbauer, Vienna, Austria). At time of investigation all participants were in stable condition and the investigator was blinded. We used Mann-Whitney-U Test and Spearman Correlation for statistical analysis.

Results: We included 63 term and late-preterm neonates (33 male and 30 female, 37 sick and 26 healthy) born after the 34th gestational week. Active ear acupuncture points were detected significantly more often in sick neonates (*p* = 0.000). Furthermore, the older the neonates were at the time of investigation, the more points were detectable (*p* = 0.000, R = 0.644). There were no significant differences in regard to birth mode.

Conclusions: This was the first blinded trial in neonates that has demonstrated that there are more ear acupuncture points detectable in sick than in healthy neonates.

### 3.17. Does Ear Acupuncture Have A Role for Pain Relief in the Emergency Setting? A Systematic Review and Meta-Analysis

#### Jan, A.; Aldridge, E.; Rogers, I.; Visser, E.; Bulsara, M.; Niemtzow, R.

Objective: Ear acupuncture might be the form of acupuncture best suited to improving emergency pain management. Our primary aim was to assess the efficacy of acupuncture in the emergency setting while secondary objectives were to explore its suitability through patient satisfaction, adverse effects, cost, administration techniques and medication usage reduction.

Methods: Seven data bases and Google Scholar were searched up to 27th April 2017 using MeSH descriptors for three overarching themes concerning ear acupuncture, pain management and emergency medicine. Meta-analysis was performed in three comparator groups of: acupuncture *versus* sham, acupuncture-as-adjunct to standard care and acupuncture (both sole and adjuvant) *versus* control to calculate the standardised mean difference and weighted mean difference for pain scores out-of-ten. 

Results: Six randomised controlled trials and two uncontrolled observational studies totalling 458 patients were retrieved after exclusions. The meta-analysis used data from four randomised studies representing 286 patients. The above three comparator groups resulted in standardised mean differences of 1.69, 1.68 and 1.66, and weighted mean differences of 2.47, 2.84 and 2.61 respectively with all figures favouring acupuncture.

Where measured, there were no adverse effects and patient satisfaction was improved. Results regarding reduction in medication use were equivocal. Significant study bias was found and Battlefield acupuncture was the most commonly used technique.

Conclusions: While study numbers are limited, ear acupuncture appears efficacious, either as stand-alone or as adjunct analgesia. It has potential benefits for its use in the emergency setting. Further studies will define this role and whether it reduces use of analgesic medications.

### 3.18. The Power of the Omega Points; Their Direct and Reverse Meaning; A Clinical Study about 250 Cases

#### Gresser, H.

The three omega points have been discovered by Dr. Paul Nogier; each of them represents an embryological tissue and its fulfilment in the physical field as well as the emotional one.

Ernst Kretschmer (a german psychiatrist 1888–1964) has shown the links between the somatic type and the psychological type.

Dr. Maurice Verdun and Dr. R.J. Bourdiol carried out from 1954 to 1970 anthropobiometric tests and managed to classify the population into four categories: «normosome», «leptosome», «pycnosome» and «athletosome».

Dr. R.J. Bourdiol made the hypothesis that these bio-psycho-morpho-typological variations were the result of an over expression of one of the three embryological tissues during the development.

We have measured the electrodermal potential of the omega points of the first 250 patients coming to our auriculotherapy consultation after the starting of this study and compared these figures to their bio-psycho-morpho-typology according to the standards of Verdun and Bourdiol.

We have found a correlation of 90% between the over expression of an omega point and its corresponding bio-psycho-morpho-type.

The clinical statements of Nogier, Kretschmer, Verdun and Bourdiol are confirmed by electrophysiological measures belonging to auriculotherapy. 

In this way we have managed to emphasize how auriculotherapy can perfectly fit into the Medical Science.

We also propose a treatment protocole to mitigate the effects of the over expression of an omega point.

### 3.19. Evolution of Auricular Nomenclature for Integrative Auriculotherapy

#### Oleson, T.

Starting in 1984, the World Health Organization (WHO) held a series of international meetings to establish a standardized nomenclature for body acupuncture and auricular acupuncture. Sessions at this auriculotherapy symposium in Singapore in 2017 strives to further finalize a standardized auricular nomenclature. The WHO meetings in the 1980’s led to a standard nomenclature for body acupuncture points. This system consisted of an alphanumeric code, the Pinyin Chinese phonetic name, the Han character for each acupuncture point, and the English translation of the Chinese names for acupuncture meridians. A two-letter rather than a one-letter abbreviation was adopted for each meridian covering the body. At a 1990 WHO international meeting held in Lyon, France, the auricular charts used by acupuncturists from Asia contrasted only slightly with the inverted fetus perspective developed by European doctors. Nonetheless, specific differences in ear point localization between Asian and Western perspectives prevented any collective international consensus. 

Rather than develop a common name for acupuncture points on the ear, an alternative approach has been to standardize the identification of the anatomical regions of the external ear where acupuncture points are located. An auricular zone system based upon the structure of the ear was originally suggested by Dr. Paul Nogier. He divided the auricle into a rectangular, grid pattern of rows and columns. While such a grid pattern is simple to use on a flat two-dimensional piece of paper, it is not as easily adaptable to the three-dimensional depths of the auricle. The curving contours of the external ear do not readily conform to the configuration of rectangular rows and columns and the Nogier auricular system did not provide a method for indicating hidden or posterior regions of the auricle. A linear grid system that used only numbers rather than letters was subsequently proposed by Winfried Wojak and Frank Bahr of Germany to designate different areas of the auricle. They noted that while the external ears may differ in the physical dimensions, but 10 auricular zones could be found on everyone’s external ears. Individual auricular zones utilized the ‘‘X’’ and ‘‘Y’’ coordinates known from classical mathematics. The proposed advantage of using only numbers was that they could be read all over the world just like postal codes. 

A very different auricular format was proposed by Oleson and Kroening in 1983. The previously identified regions of the auricle, such as the curving structures of the helix, antihelix, and concha, were further subdivided into smaller auricular zones. Single letter abbreviations for these anatomical regions were switched to two letter abbreviations after the 1990 WHO meeting, such as HX for helix and AH for antihelix, which was further subdivided such as HX-1, HX-2, etc. Subsequent auricular nomenclature systems developed by Liqun Zhou of China, Marco Romoli of Italy, and David Alimi of France also developed curving spiral arrangements of auricular zones. Further concurrence regarding these different nomenclature systems remains unresolved. 

### 3.20. Cautery in Auriculotherapy

#### Nogier, R.

Dedication: To Marco Romoli who studied deeply the roots of auriculotherapy.

Introduction: Practised during numerous generations by a large number of healers and quacks, ear cauterisation was forgotten in today’s practice, which is quite a pity. It is true that medico-legal issues in connection with this technique, frighten many practitioners. However, cauterisation is irreplaceable in auriculotherapy, in view of its high efficiency to fight certain pain: sciatica, cervico-brachial neuralgia. 

History: Cauterisation belongs to medical patrimony of the Occident. Hippocrates, four centuries before JC, recommended cauterisations in order to treat certain pathologies. One of his latest aphorism (section VII, n°87) precises that diseases which cannot be cured by medicine, can be cured by surgery. Those which are not curable by surgery, can be cured by fire (cauterisations). Those, which are not curable by fire cannot be cured at all. 

We can find this practise all along the history of medicine in Mediterranean sea: Avicenna, Albucasis, Ambroise Paré, P.F Percy. Indications for cauterisation were large: hemostasis, wound cleaning, abscess treatment. It was also used to fight pain. In this case, cauterisation was applied loco dolenti. 

For migraines and dental neuralgias, due to the fact that it was difficult to cauterise the scull or the jaw, it seems that cauterisations were done on the upper part of the ear in case of a migraine and on the backside of the ear in case of dental pain.

Cauterisation on the ear for sciatica: It is however strange to note at the XVIIth century the existence of ear cauterisations for sciatica in Europe. In the XIXth century, professor Malgaigne (Paris), estonished by the results obtained by the healers, practiced himself cauterisations in the hospital of Saint-Louis. 

Brown-Séquard, the founder of neurology appreciated this technic in a lesson presented the 7th of November 1866 at the occasion of an opening session of medical lessons at the Harvard university.

One century later, Paul Nogier is confronted to two patients cured from sciatica through cauterisation on the ear realised by a quack from Marseille, Madame Barrin, who herself was a descendant from a Corsica family. He also tried out himself to apply the treatment of cauterisation with succes. Starting from this cauterisation, Paul Nogier will construct a veritable method, which he called «auriculotherapy».

### 3.21. Documented Tracking of the Immediate Effect of Auricular Acupuncture in Specific Cases of Neurology, Endocrinology, Gynaecology and Otolaryngology

#### Szechnyi, I. 

Background: In case of locomotor problems, if the applied auricular acupuncture therapy is successful, the result becomes apparent immediately. In case of functional diseases the treatment may prove to be very spectacular: symptoms and complaints cease to exist, and the results are later confirmed by lab tests and instrumental examinations. 

Auricular acupuncture had an immediate effect in the following cases:

Treatment of mammary secretions and normalisation of extremely high PRL level;

Treatment of 5.1 cm and 3.3 × 4.4 cm ovarian cyst;

Treatment of 9 × 11 cm uterine fibroids with auricular acupuncture;

Treatment of headache;

Treatment of the loss of hearing of a 3-year-old boy with auricular acupuncture and laser;

Treatment of hyperthyroidism with auricular acupuncture.

Methods: In each case the healing process is tracked and documented according to the practice of western medicine (lab, MRI, etc.) and Chinese medicine (meridian-diagnostics).

Results: Even after the first ear acupuncture treatment the symptoms and complaints either completely ceased or significantly reduced. By using meridian diagnostics the improvement in the balance of the meridians is immediately apparent in the images then, somewhat later in time, the lab tests and instrumental examinations according to western medicine confirm the success of the treatments.

Conclusion: The immediate successful effect of auricular acupuncture may not only be experienced in the case of locomotor disorders, but also in cases presented above, documented both according to western and eastern medicine. 

### 3.22. A Look at Subcortex through the Prism of Polyvagal Theory

#### Wu, H.

Subcortex, a point found on the medial surface of the antitragus, is poorly understood yet of great therapeutic importance. One senior acupuncturist, Dr. Li Chun Huang OMD (China), has uniquely divided this point into a triangle: a neuro; vasocoronary; and digestive subcortex. Inspired by new ideas about the autonomous nervous system from Dr. Stephen Porges, Polyvagal Theory. A Californian acupuncturist attempts to shed some light onto this triangle to understand it’s physiology so to better to use it in Stress related disorders such as PTSD.

### 3.23. Significance of Auricular Acupuncture for Treating Some Skin Diseases in Children

#### Luzina-Chju, L.; Luzina, K.

Allergic skin diseases constitute persistent and significant problem for medical science. Being a popular subject of different scientific research this kind of skin diseases is still in the focus of attention and the rate of its sickness is growing.

Nowadays there exist numerous modern medicaments for treating different allergic skin diseases. They can help relieving the symptoms but it is insufficient to remove the cause of the illness.

From the point of view of the traditional Chinese medicine, skin and allergic diseases are considered signs of other disorders. They can be caused by

1. immune dysfunctions;
2. infections, fungi, bacteria, viruses, helminthes;
3. food allergies;
4. problems with the organs of digestion;
5. metabolic disturbances;
6. psycho-emotional dysfunctions;
7. Experience shows that endocrine disorders are at the root of many skin diseases. A baby may have skin problems because the mother suffered from hormonal disorders before and during her pregnancy;
8. The hereditary factor is very important too;
9. And as we all know, the Chinese associate skin problems with the channel related to the lungs.

In our experience, children suffering from acute skin conditions may often have

1. a significantly increased level of fructosamine;
2. decreased levels of thyroxine and cortisol;
3. or high titers of immunoglobulin E. (IgE).

They may suffer from a hypo function of the thyroid and adrenal cortex. In some cases ultrasound examinations show an enlarged pancreas. 

Auricular diagnostics was conducted as a the most effective method that allows to get the most objective results. The auricle was examined and the following characteristics have been taken into account: the form, the color of the skin, intensity of vascular pattern, presence of various morphological elements (spots, scars, peeling etc.) and their location. Palpation was also applied to reveal sensitivity and zones of hyperalgesia. On the basis of the diagnostics the conclusion was made that the zones of high sensitivity threshold and zones of altered outer indications revealed functional depression of kidneys, lungs, endocrine glands. According to the obtained results the following points were stimulated: 

Shen-Men—55, 22—internal secretion glands, Zero Point, 34—cerebral cortex, 101–lungs, –Allergy, These points help to calm down the nervous system and relieve itching

55—Shen Men (Spirit Gate, Divine Gate)

We applied this point because it alleviates stress, pain, tension, anxiety, depression, insomnia, restlessness, and excessive sensitivity.

22—Endocrine point (Internal Secretion, Pituitary Gland)

As you know the pituitary is the master gland controlling all other endocrine glands. So we used it because it has anti allergic, anti-rheumatic, and anti-inflammatory effects. In TCM treatments, it reduces dampness and relieves swelling and edema

Point Zero (Ear Center, Point of Support, Umbilical Cord, Solar Plexus). This point supports the actions of other auricular points and returns the body to the idealized state which was present in the womb.

Point Zero serves as the ’autonomic brain’ that controls visceral organs through peripheral nerve ganglia.

34—Cerebral point (Master Omega, Nervousness, Neurasthenia, Worry).

We selected this point because its stimulation diminishes nervous anxiety, fear, worry, lassitude, dream-disturbed sleep.

101—Lung.

This point controls breathing, regulates body temperature and promotes circulation of oxygenated blood. It showed efficiency in treating edema, night sweats, skin disease/irritation and laryngitis. Since lung and large intestines are intimately related we used it for treating enteritis and diarrhea. Allergy point

This point leads to a general reduction in inflammatory reactions related to allergies, rheumatoid arthritis and asthma. So we applied it for the elimination of toxic substances, the excretion of metabolic wastes and treatment of anaphylactic shock.

We can come to the conclusion that:

1. Auricular acupuncture is an efficient approach to the treatment of skin and allergic diseases in children. It can quickly relieve and often remove such symptoms as itching, redness, weeping, crusts, peeling, edema, skin inflammation etc.
2. Auricular acupuncture therapy:
  a) strengthens the immune system,
  b) stimulates blood circulation,
  c) relieves stagnation,
  d) improves local metabolic processes,
  e) regulates the vegetative nervous system, the digestive and the endocrine system,
  f) normalizes the function of the pancreas, liver and lungs,
  g) alleviates allergic conditions,
  h) helps to get rid of infections, fungi, bacteria, viruses, helminthes.
3. Using auricular and corporal acupuncture is safe. Acupuncture can be applied as an alternative or as a complement of the medication treatment.
4. Acupuncture helps to improve the children’s general well-being and has a positive effect on their psycho-emotional condition. It helps to decrease nervousness and anxiety. Children become calmer and more relaxed.

### 3.24. Transcutaneous Auricular Vagus Nerve Stimulation (ta-VNS) Modulates Rest Pain of Lower Limb Arteriosclerosis: A Case Report

#### Huang, F.

Background: Rest pain is the main symptom in the middle and later periods of the lower limb ischemic diseases such as arteriosclerosis obliterans (ASO) and thromboangiitis obliterans (TAO). The incidence of ASO is about 10%, and increased with age, over 70 years old the incidence up to 15–20% [1,2], and patients who with diabetes have a higher incidence of 90% [3,4]. Long-term state of rest pain makes patients up all night, and limb amputation makes them lose labor ability, even decreases heart rate and cardiac arrest due to severe pain, affects their physical and mental health and life quality seriously, increases health-care costs and the financial burden to families and societies. Treatments of rest pain mainly via oral and intramuscular analgesic, but carefully used because of the drug resistance, drug dependence and the adverse reactions for patients.

Methods: We treated one case of lower limb arteriosclerosis occlusion by transcutaneous auricular vagus nerve stimulation (ta-VNS) with 20 min.

Results: The VAS score of pain from 4 fallen to 2. Scanned and compared the patient before and after treatment with the health people with normal resting state of brain function used fMRI technology. We found that before the treatment, anterior cingulate central activated obviously in patient with rest pain compared to the health one, but after the ta-VNS treatment, the activation area decreased significantly. Conclusions: This prel*iminary c*ase study related that ta-VNS could adjust the networks of central brain and that maybe the mechanism of relieve pain.

1. Pasternak, R.C.; Criqui, M.H.; Benjamin, E.J.; Fowkes, F.G.; Isselbacher, E.M.; McCullough, P.A.; Wolf, P.A.; Zheng, Z.J.; American Heart Association. Atherosclerotic Vascular Disease Conference: Writing Group I: Epidemiology. *Circulation* **2004**, *109*, 2605–2612.
2. Nehler, M.R.; Wolfor, H. Natural History and Nonoperative Treatment of Chronic Lower Extremity Ischemia. In: *Vascular Surgery*, 6th ed.; Rutherford, R.B., Ed.; Saunders: Philadelphia, PA, USA, 2005; pp. 1083–1094.
3. Estes, J.M.; Pomposelli, F.B., Jr. Lower extremity arterial reconstruction in patients with diabetes mellitus. *Diabetic. Med*. **1996**, *13*, S43–S47.
4. Gavin, A.; Stess, R.M.; Goldstone, J. Prevention and treatment of foot problems in diabetes mellitus: A comprehensive program. *West. J. Med*. **1993**, *158*, 47–55.

### 3.25. Gamma-Frequency Transcutaneous Auricular Vagus Nerve Stimulation: A Promising Therapy for Alzheimer's Disease

#### Yu, Y.T.; Rong, P.J.

With the acceleration of aging population, the disable elder due to Alzheimer’s disease (AD) increased year by year, causing heavy burden to the society and families. “China Brain Project” also focus on the AD. However, the existing and newly developed drugs can slow down the process the AD, but were unable to effectively reverse it. Some evidences have confirmed that Vagus Nerve Stimulation (VNS) is effective for AD. Ten AD patients were recruited in a small-sample VNS clinical trial, in the first 6 months of treatment, patients’ cognitive function has been significantly improved. Another small-sample VNS clinical trial also observed that after 2 weeks of implantation, all patients had a significant increase in attention; 3 patients with language disorders were significantly improved after 3 months of implantation. The afferent projection branch of the vagus nerve at the auricular concha of mammals allows the transcutaneous auricular VNS (taVNS) developed based on this rationale years ago, and proven to have comparable efficacy to classic VNS. In April 2012, Jorge J. Palop, a neurobiologist of Gladstone Institute in California, published a paper in *Cell*, telling that restore the gamma wave can improve the memory of mice models of AD. In December 2016, Li-Huei Tsai of MIT published a paper in *Nature*, announcing that scintillation stimulation with implanted optogenetic device in gamma frequency (40 Hz) can significantly reduce the AD mouse brain β-amyloid protein content and Tau protein concentration. Thus, gamma frequency (40 Hz) may be the key of all these. Since optogenetic stimuli will be converted into electrical signals in the brain, it is speculated that gamma frequency (40 Hz) electrical stimulation will have a greater advantage in AD treatment. TaVNS can directly stimulate the brain via the vagus nerve without surgery, which can avoid the risk of postoperative infection. Therefore, gamma frequency taVNS is a very promising AD treatment.

### 3.26. Transfloral Acupuncture

#### Wirz-Ridolfi, A. 

In any patient the extension of his actual electromagnetic field is decisive for his state of health as well as for the effectiveness of any therapeutic measure. With the innovative method of Transfloral Acupuncture it is possible to improve the energetic level of patients considerably within seconds.

Most foci (which are obstacles for diagnosis and treatment) are dental foci.

Teeth, even if they don’t need treatment by a dentist yet, can present a focus causing a considerable loss of energy for the patient. This loss of energy reduces his capability to react to any treatment as well as his immune system.

According to the research of Prof.Frank Bahr and Dr.Heike-Dorit Schmid every tooth is in resonance to a flower essence (Bach, Californian flower essences or Australian bush flowers). By means of the Vascular Autonomous Signal (VAS) and the Bahr detector (a combination of a 3-Volt hammer and a black and white hammer) all the teeth are controlled and the corresponding flower essence is chosen. A drop of the floral essence is given orally and another drop is placed on the ear lobe of the patient at the place of the tooth found to be a focus. Now a permanent needle is inserted through the floral essence into the ear lobe. This manouevre I call “Transfloral Acupuncture”, needling through the flower.

In the period between 24 November 2014 and 10 November 2015 the energy field of 168 patients (100 female and 68 male) was established in 616 measurements before and after each treatment. The average improvement was 59, 35 centimeters.

By this improvement of the energy field the patient feels more energetic, is more resistent to infections and reacts better to any treatment given afterwards.

### 3.27. Transcutaneous Vagus Nerve Stimulation: A New Type of Auricular Acupuncture to Treat with Depression

#### Li, S.

Auricular acupuncture therapy is a crucial treasury of traditional Chinese medicine. In terms of the national standard of auricular name and position in the People’s Republic of China, acu-points of internal organs, such as heart, liver, kidney and spleen, are located in auricular concha, where the only area in our body distributed with auricular branch of vagus nerve. Our team has spent more than ten years to study transcutaneous vagus nerve stimulation (tVNS) to treat with depression from animal experiments to clinical trials. Our functional magnetic resonance imaging results demonstrated that the efficient of tVNS was associated with increased functional connectivity between the default mode network and precuneus and orbital prefrontal cortex, an important network in the brain known to be altered in depression [1]. tVNS can inhibit the reduction of the open- field scores and increase of plasma cortisol and adrenocorticotrophic hormone in unpredictable chronic mild stress rat models [2]. Then we attempt to explore the effect of different frequency electric stimulation and observe the expression of c-Fos and ∆FosB in NTS, LC and DRN in depressive rat models. We will detect the change of some chief elements related to depression in hippocampus, amygdaloid nucleus and prefrontal cortex by real- time and Western blot respectively from the expression of mRNA and protein level. At last, we will study the effect of apoptosis and regeneration in hippocampus of tVNS and expect to probe the mechanism of tVNS to improve depression from cellular level. 

The studies were supported by Beijing Municipal Science & Technology Commission (Z161100002616003).

1. Fang, J.L.; Rong, P.J.; Hong, Y.; Fan, Y.; Liu, J.; Wang, H.; Zhang, G.; Chen, X.; Shi, S.; Wang, L.; et al. Transcutaneous vagus nerve stimulation modulates default mode network in major depressive. *Biol. Psychiatry* **2016**, *79*, 266–273.
2. Liu, R.P.; Fang, J.L.; Rong, P.J.; Rong, J.; Zhao, Y.; Meng, H.; Ben, H.; Li, L.; Huang, Z.X.; Li, X.; et al. Effects of electroacupuncture at auricular concha region on the depressive status of unpredictable chronicmild stress rat models. *Evid. Based Complement. Altern. Med*. **2013**, *2013*, 789674. doi:10.1155/2013/789674.

### 3.28. Neurophysiological Basis of Auriculotherapy for Perioperative Pain Management

#### Chelly, *J.*

Background: Auriculotherapy otherwise known as auricular acupuncture is an effective complement to modern pharmacotherapies to treat numerous pathologic conditions. It is considered a safe and cost effective approaches to improves outcomes, especially when used for the treatment of acute and chronic pain including low back pain, pain associated with trauma and surgery, fibromyalgia, neuropathic pain, myofascial pain, and even opioid addiction in the adult and pediatric population. 

Method: This presentation based on a systemic review of the literature to provide evidence supporting the objective use of Auriculotherapy /Auriculo-acuponcture for the management of acute and chronic pain with a special focus on evidence supporting the use of the traditional Chinese/German Auriculo-acuponcture and also the use of the French scientific Auriculotherapy. 

Results: Based on the review of randomized of clinical trials (with or without a control group), case reports and series, it is established that various techniques have been proposed for the management of pain. Although many of them claimed to be efficacious, at the present time it is very difficult to establish the relative efficacy of each technique. The same apply to approaches and protocols. Used to treat a given pathologic condition. **Conclusions:** Additional are required to establish which technique, approach, and protocol should be chosen for the management of pain using Auriculotherapy.

### 3.29. A Detailed Comparative Analysis of the Effects of DC Micro-Current Point Stimulation (MPS) on the Autonomic Nervous System (ANS) when Applied to Battlefield Acupuncture (BA) Protocol of a N = 8 Patient Sample Size with a History of Pain

#### Armstrong, K.

Background: A detailed comparative analysis of the effects of DC Microcurrent Point stimulation (MPS) on the autonomic nervous system (ANS), when applied to Battlefield acupuncture (BA) protocol of a *n* = 8 patient sample size with a history of pain. 

Methods: Evaluations entailed a Standard Protocol baseline NPRS (VAS) pain scale, Cortisol and a baseline status of 27 ANS functions, all repeated pre-post to electro-therapy on a *n* = 8 sample size with a history of pain. 

Results: The ANS response of a n = 8 patient sample with chronic pain electrical nerve stimulation Microcurrent Point stimulation reflected a statistically significant pre-post improvement in seven of the 29 markers collected: pain reduced 2.0625 points or 63% [95% CI (1.2745, 2.8505; *p* = 0.0001], HRV improved 662.375 points or a 42% [95% CI (−1273.675, −51.075); *p* = 0.037), HF-Vagal tone improved 231.25 points or a 56%, [95% CI (−430.42, −31.58); *p* = 0.029]. Exercise tolerance-SDANN increased of 9.500 points or 22% [95% CI (−16.747, −2.253); *p* = 0.017], RMSSD- Parasympathetic activity improved 14.000 points or a 38% [95% CI (−23.202, −4.798); *p* = 0.009], Stress: reduced 39.125 points or 27%, [(95% CI (1.945, 76,305); *p* = 0.042], and PTGi-Cardiac marker improved 21.5125 points or a 48% [95% CI (−35.441754, −7.5832461); *p* = 0.008)]. 

Conclusion: The positive results in this study could will help establish the validity of MPS applied to BFA protocol for other pathologies that can be impacted by the sympathetic nervous system activation on the body.

### 3.30. The Use of Auriculotherapy for Cardiac Arrhythmias: A Systematic Review

#### Mok, M.; Suen, L.; Tan, J.Y.; Xie, G.

Background: Patients with paroxysmal type of cardiac arrhythmia often experience poor quality of life due to the low successful rate of the mainstream treatments in controlling the disorder. Being one of the complementary health approaches, auriculotherapy was found to be a safe treatment modality which may benefit patients with cardiac arrhythmias. This systematic review therefore evaluates the effectiveness of auriculotherapy on patients with this problem.

Method: Literatures were searched through Pubmed, Embase, CENTRAL, China National Knowledge Infrastructure (CNKI), WangFang Database, and VIP Database using relevant keywords and mesh terms. All articles were screened by title and abstract to identify relevant clinical studies.

Results: A total of 341 relevant articles were identified from 1990 to 2017. Eight clinical trials involving 2050 participants were included Subjects with premature atrial contraction, premature ventricular contraction, sinus arrhythmia and/or sinus tachycardia, were included in most of these trials. The results suggested beneficial effects of auriculotherapy on the reduction of heart rate and/or arrhythmia episodes compared with the controls. However, generalizability of the findings was limited because of significant methodological flaws, such as inadequate information regarding the randomization process, blinding, and allocation concealment.

Conclusion: The findings of the systematic review indicated a plausible effect of auriculotherapy on patients with cardiac arrhythmias. The implications drawn from these studies put some clues for future high-quality trials so as to determine the effectiveness of auriculotherapy on cardiac arrhythmias. A more detailed report of the systematic review and bias assessment of these studies will be presented in the symposium.

### 3.31. Comparative Examination of the Ear Acupuncture Points (NADA/Battlefield) in Light of Western Medicine (Lab, Instrumental) and of Chinese Medicine (meridian diagnostics)—Randomized, Placebo-Controlled, Double Blind Research

#### Szechenyi, I. 

Background: Auricular acupuncture/acupuncture has been often attacked on grounds that it makes no difference which points are stimulated by needles, the effect will be the same, and even in an optimal case ”only a placebo” effect is apparent. 

To investigate the importance of localization and the placebo effect we chose to use the internationally recognized 5-point NADA (Smith) and 5-point Battlefield (Niemtzow) protocol. 

Methods: Randomized double blind placebo control (RDBPC) studies, (the second one only RDBP). 44 persons participated in our first research, while 110 persons in the second one. 

Results: Both in the group which was treated with needles and in the NADA group which was treated with laser the values of the PRL, CORT and MeriDiM significantly reduced. At present, we are performing a comparative analysis between the NADA and the Battlefield protocol with MRI.

In the case of the Battlefield group, the changes of the PR and CORT level stagnated, while the measured values of the MeriDiM^®^ decreased to the greatest extent.

Conclusion: Comparing the effect of the specific NADA 5-point treatment (either needle or laser treatment) with the non-specific effects of Battlefield treatment, based on the PRL and CORT response levels of the human body the points of NADA have an immediate significant stress-reducing effect, while the Battlefield points don’t. 

## 4. Workshops 

### 4.1. Battlefield Acupuncture Training Across Clinical Settings—Initial Results & Lessons Learned

#### Pock, A.; Niemtzow, R.

Background: Within the United States, the need for safe, effective, and efficient management of acute and chronic pain is becoming a health-related priority of national proportions, particularly when viewed in the setting of a growing epidemic of opioid dependence and overuse.

Objective: To evaluate a large scale, $5.4 M multi-disciplinary, multi-site teaching program designed to facilitate the effective implementation of the Battlefield Acupuncture (BFA) technique at 26 different medical facilities across the U.S. Department of Defense and at 21 different Veteran’s Affairs Hospitals. 

Intervention: The BFA technique was taught to nearly 3000 medical personnel over an 8-month period. Only 44.3% of the trainees were physicians; the rest were a combination of nurses, dentists, medical technicians and other health related personnel. Less than 1% had any prior training in acupuncture.

Results: BFA is an effective technique that can be used for the amelioration of acute or chronic pain in a wide variety of clinical settings. The technique can be easily taught to a wide range of health professionals—to include those with no prior training in auriculotherapy. Even more important the training can be accomplished in a short period of time (approximately 4 h) with reproducible and sustained results. 

Conclusions: Implementation of a large scale, nation-wide, BFA training program is a feasible adjunct to improving the health and well-being of a broad range of patients. There are, however, some lessons to be learned, which will be the focus of this presentation.

### 4.2. New Insights into the Autonomic Nervous System Using Beat to Beat Methodology Monitoring

#### Lafitte, M.; Nageshwar, S. 

Following an approach of empirical dynamic modeling which makes no assumptions about the underlying relation between cardiac dynamics and autonomic changes, we extract beat-to-beat descriptors of the Autonomic Nervous System (ANS) from the variability of R-R intervals.

Short and long term interactions are observed; respectively, the sympathovagal balance trajectory and the autonomic dysfunction percentage are calculated. Though the sympathetic (Sp) and parasympathetic (pSp) are generally thought to be antagonists, the descriptors reveal a complex interplay involving co-activations and delayed inhibitions. Supposedly vagal maneuvers, e.g., rhythmic deep breathing or the Valsalva maneuver, tell transient pSp predominance and coupled triggering of both autonomic subsystems, while retaining an overall vagal tone. Combined heat and tilt stress induce heart rate changes, which discriminate the invariance of the pSp while agreeing with the mechanisms of a Sp maneuver. Recovery from the tilt test further illustrates the homeostasis that underpins the interactions between the counterparts of the ANS. A thermodynamic outlook is additionally provided, translating activities into energy transfers. Moreover, the cold pressor test and yoga exercises are studied in relation with their autonomic imprint; the latter for the impelled pSp reinforcement and the former for the pure Sp bursts it kindles. Turning to long-term assessments, we further investigate the differences in populations undergoing degenerative diseases, e.g., diabetes mellitus, as related by the evolution of the impairments at the level of the ANS.

These new and updated insights into the ANS substantiate knowledge at the gross physiological level while overhauling the particulars. The clinical advantage of a repetitive measure of autonomic dysfunction and of monitoring the sympathovagal balance trajectory is addressed.

### 4.3. Essentials of Auriculotherapy

#### Oleson, T.

This half-day workshop on the *Essentials of Auriculotherapy* presents effective clinical approaches for the detection and treatment of ear reflex points that are used for the alleviation of chronic pain, substance abuse, and stress-related disorders. The focus of this course provides fundamental knowledge of the anatomical regions and anatomical landmarks on the auricule that assist practitioners in localization of specific ear reflex points. The somatotopic correspondence of health disorders in the physical body are identified with the inverted fetus pattern represented on the external ear. The theoretical understanding and the anatomical localization of Chinese ear acupuncture points as compared to the European systems of auriculotherapy will be described. Specific auricular reflex points which will be highlighted include master points on the external ear, musculoskeletal ear points, internal organ ear points, and neuroendocrine ear points. Didactic presentations, clinical demonstrations, and hands-on training will facilitate a deeper understanding of auriculotherapy microsystem that is of clinical benefit to all practitioners in this field.

### 4.4. How to Use Psychological Points at the Ear with Exactly Fitting Flowers

#### Wesemann, C.

This lecture and workshop will show the fascinating world of psychological barrier points at the ear with exactly fitting flowers for people with psychological problems. Patients with an unstable, labile mental state with acute symptoms are unable to look behind the acute problems, which are the result of old suppressed hurt in their childhood or in some difficult parts in their life. Here we are finding old sense of guilt, animosity, rage, anger or harassment at work and with the help of the psychological barrier points and the belonging flowers we are able to bring patients back in an amazing balance. So the acute symptoms are vanishing.

### 4.5. Auriculotherapy in Neurology Workshop—Part 1

#### Stanton, G.

This workshop will focus on the use of auriculotherapy in a variety of neurological conditions. The approach will strive to be evidence-based. By introduction, auricular acupuncture mechanisms will be reviewed including research-based neurophysiological and other current physiological theories of auricular acupuncture effect.

Attention will then be given to general principles of neurological diseases and syndromes. The neurological examination itself will be briefly reviewed. Following that, certain specific neurological disorders will be discussed, emphasizing those where evidence-based acupunctural literature has been published. Examples include facial paralysis, cerebrovascular disease, movement disorders, peripheral neuropathy, epilepsy, and sleep disorders. Of note, pain syndromes will be excluded from this presentation.

Although comparative auriculotherapeutic diagnosis and treatment approaches will be discussed, in general the clinical method emphasized will be that of the inter university diploma *(diu)* program of auricular acupuncture of the university of paris xi.

Three objectives of this workshop will be:

1. Review acupuncture mechanisms underlying auriculotherapy in neurology.
2. Understand fundamental principles of neurological syndromes and disease, and of the neurological examination, as a guide to neurological diagnosis and auricular acupuncture treatment.
3. Review specific neurological diseases and their auricular acupuncture treatments, with emphasis on an evidence-based approach wherever possible.

The target audience will be physician and non-physician acupuncturists and auriculotherapists who desire to learn more about the use of auriculotherapy in the treatment of patients with neurological disorders, excluding pain syndromes.

### 4.6. A Primer on the Phases & Recognizing the Vascular Autonomic Signal: The Key to Successful Clinical Outcomes

#### Chalmers, J.

Dr. Paul Nogier, ‘The Father of Modern Ear Acupuncture’ is well known for his anatomical mapping of the homunculus, “the Man in the Ear”, and for his term “*Auriculotherapie*” describing his approach to treating ailments via the auricle. Lees well known is Nogier’s discovery of a perceptible change in the patients pulse in response to a small stimulus. He called this the Vascular Autonomic Sign (VAS) and this was the key to his development of Auriculomedicine. 

When using differential electrical detection, the gold standard for objective identification of auricular points, the practitioner will note that active points present in a very small (within 2 mm diameter) discrete zone. Given this fact, it stands to reason that practitioners must have a valid method to locate active points. 

A 2014 survey of Ear Acupuncture practitioners in the UK indicated that more than 50% did not think it was necessary to have a valid technique, such as palpation for tenderness, or electrical detection to identify active points. The implication was that referring to charts was sufficient. 

TCM colleges in the West teach an Ear Acupuncture module in their courses with the intention of it being mostly utilised as a supplementary treatment to TCM body acupuncture rather than a stand-alone, patient-specific first line treatment. Point selection training is usually limited to ahshi points detected by palpation and electrical detection. Credence is usually only given to Dr. Paul Nogier’s original cartography, unfortunately without mention of his further discoveries.

Doctor Paul Nogier taught how to use the VAS to locate and precisely identify active auricular points. Although this method can be viewed as subjective it has proved to be very accurate, more convenient and patient friendly—bringing a whole new dynamic to auricular treatment. 

For the continued growth of Auriculotherapy and Auriculomedicine as a patient specific, therapeutic approach, it is essential that the practice-changing discoveries used in Nogier’s later development of Auriculomedicine, be brought back for use in Auriculotherapy. The most important of these include the location of points on the ear using the VAS, the Phases, treatment of Laterality disorders and the clearance of Toxic Scars. These skills are easily learnt and can make quantum changes to clinical outcomes.

Auriculotherapy training courses emphasising Dr. Paul Nogier’s discoveries should be made available to suitably qualified practitioners worldwide.

### 4.7. Light & Colours: The Vasculo-Autonomic Signal & Healing

#### Nogier, R.

In auriculotherapy, we use the photonic stimulation on the skin and on the ear to trigger the VAS phenomenon. Takink the radial pulse, we can measure the photo-perception with simple filters and a little torch lamp. We can use also sophisticated devices as the microlightR. This technic is very useful to make a diagnosis and to choose the ear points that we must treat.

The white light test: It is the basic test. The aim of the test is to appreciate the quality of the VAS. Technicaly, we project a light on the skin of the patient and immediately, we feel a VAS reaction. We repeat this illumination several times. Normaly the VAS phenomenon is not exhaustible. If the VAS is exhaustible, we must research a dental focus or a perturbating scar. If there is no VAS, we must research a burn-out or a cancer.

The photogram: We study the VAS response when we light the skin with coloured lights.Normaly each coloured stimulation trigers a VAS reaction. 

When the white light test or the photogram are affected, we must research on both ears some active points and teat them. Normally these ear points will normalize the tests. 

### 4.8. Auriculotherapy in Neurology—Part 2

#### Stanton, G.

This 2 h workshop will explore auricular acupuncture as an evidence-based intervention in a broad variety of neurological applications, with the notable exception of pain management. The purpose of the presentation will be to review the potential scope of auriculotherapy in neurology. The clinically oriented discussion will focus on a variety of neurological diseases and syndromes for which there have been published reports of auricular acupuncture interventions.

### 4.9. Genomic Acupuncture Precision Medicine—Introductory Outline Innovated for Auricular Application. 

#### Johnson, S.

Originated Genomic Acupuncture E-Energy Model Precision Medicine©

1. G.Ac is a Macro-Micro Chinese Acupuncture Medicine System also applicable for Auricular Diagnosis & Treatment. The perfect East West Medical Bridge.
2. G.Ac is the bespoke, unique individuals E-Energy DNA Gene Make up Genome i.e., Origin/Cause. G.Ac protocol influences regulation of positive gene expression.
  - It is not based directly in Symptoms/Effect, defective gene or manifest illness but these are treated/benefit indirectly through my G.Ac E-Model System Concept yet unknown to modern TCM/Western Medicine.
3. G.Ac demonstrates clinical concordance and auricular medicine concordance of.
  - Energy, Cell DNA Gene, Long Bone, Auricular, Embryo, Adult concept stages, the Yin Yang Expression of Taiji Philosophy all as one holism.
4. I am invited as stream Co-Chair and Speaker at the World Gene Conference Macau November 2017 where I deliver my full G.Ac Discovery Abstracts and Presentations for the first time to a worldwide professional audience.
5. Korea and China laboratory tests show acupuncture point needling effects gene expression positively, 799 genes in one test.
6. Sub-Particle Atomic Physics recognises charge, spin, vibration ‘energy’ but has no model or what the energy driver is for it. Neither do Higgs-Boson or Western Medicine Science.
7. My G.Ac is a missing model in The Code of Life.

Completely Self-Funded through successful independent private practice.

### 4.10. The Treasure in Auricular Diagnosis and Therapeutics

#### Aung, S.

Auricular Diagnosis is one of the most important diagnostic methods in Traditional Chinese Medicine (TCM). Since the ear is considered to be a microsystem of the human body due to its anatomical association with the inverted human embryo, medical diagnosis may be obtained through the ear by examining the physical features. Signs and symptoms such as colour changes, formation of nodules, visible appearance of blood vessels, moles, and auricular lines are vital in the procurement of the diagnosis of a patient, capable of diagnosing problems with 60–80% accuracy. The anatomical connection between the ear and parts of the body goes both ways; pathological problems elsewhere in the body can not only be diagnosed via the ear, but also treated. Pathological points are detected by an electrical detector, but can also be located by the *auriculocardio reflex* (first described by Paul Nogier), the sensitivity and pain reaction to applied pressure. Giving acupuncture to the corresponding ear part will treat the problem area in the body. The advantage of treating via the ear is convenience and easy accessibility—therefore, easy adjustment of needles and placement of ear patches. Ear patches can mediate pain, anxiety, tension, and provides constant stimulation to the ear. Hence, auricular diagnosis is vital in Traditional Chinese Medicine for its reliability in diagnosis and its therapeutic value in providing treatment, particularly for addictions or behavioural problems and for follow-ups.

### 4.11. ECIWO Auricular Diagnosis—the Greater System of Acupuncture Diagnosis

#### Ang, T.T.

One of the most complex and difficult tasks in practicing Acupuncture is the diagnosis. Using traditional methods of diagnosis, it can be too complex to begin with, let alone mastering the techniques. Furthermore, it can be a daunting and time consuming task to explain the TCM concepts to patients, as well as to convince them to accept the diagnosis findings because sometimes there lacks concrete evidence or readings of any form.

The Embryo System’s Auricular Diagnosis is easy to master and apply. It is also easier to explain to patients and patients are more willing to immediately accept the findings.

### 4.12. The Usefulness of Auriculodentistry in the Daily Practice of the Art of Dentistry

#### Vulliez, C.

The mouth is fundamentally a barrier between the human being and the outside world. The tooth is in direct neurological contact with the brain. The tooth preserves the the entirety of our genetic signaling capacity for many years. Auriculodentistry allows us to detect an auricular dental zone of interest. Diagnosis is based on the reflex of Nogier, which is perceived while palpating the radial pulse. All of this is accomplished somewhat in the manner of a computer keyboard, on which we input stimulatory information about the osteogingivodental organ, and then receive information on a “computer screen” in the form of the radial pulse reflexive phenomenon. This method allows detection of pathological information emanating from a part of or the entirety of the osteogingivodental organ. The temporomandibular joint is one of the important exteroceptors of posturology which must be examined in auriculodentistry. Dental oscillations occur frequently, be they dento-dental, dento-facial, or dento-peripheral, but are difficult to diagnose without the help of auriculodentistry. Treatment is based on the puncture of the skin of the body or of the ear. Such punctures may be direct as with a needle, or indirect via the use of specific frequency stimulations established in advance, based on the diagnostic findings. We will study numerous clinical cases, documented by photographs of the disorders involved. We will also carry out practical workshop demonstrations on volunteers among the participants.

### 4.13. Rapid Auriculotherapy Treatment for Headaches Caused by Mild Traumatic Brain Injury

#### Pock, A.

Dr. Pock (on behalf of Dr. Niemtzow) will discuss the outcomes of a randomized exploratory study designed to evaluate two different approaches to using acupuncture (i.e., traditional Chinese acupuncture vs. a rapid auricular protocol) for treating headaches associated with traumatic brain injury (TBI).

### 4.14. Finding Effective Remedies in Clinical Practce by Auriculohomeopathy

#### Lee, C.

Objective: Finding Effective Remedies in Clinical Practice by Auriculohomeopathy. This involves putting liquid homeopathic remedies on specific ear zones to elicit a clinical response.

Methods: 220 patients from general practice between June and December 2011 were sampled. This included earache, headache, toothache, abdominal pain, vertigo, skin allergies etc. Patients were assessed on the intensity of their dominant presenting symptom (e.g., pain or discomfort) from a medical and homeopathic (Boenninghausen) perspective. Symptom intensity (e.g., pain) of each case was assessed on a Visual Analogue Scale of 1 to 10 (0 = no pain 10 = maximum pain). Homeopathic remedies on a cotton bud were then applied onto specified zones of the ear. The V.A.S. was reassesed 3 min later. Commonly 2 to 3 remedies were used. A positive response was determined when the change in intensity was greater than 2/10. A negative or equivocal response was determined when the change was 1/10 or 0. 

Results: For each condition, the effective & non effective remedies in each case were recorded. This was also recorded in an overall chart to show the number of times an effective and non effective remedies were tried in each diagnosis. 

Conclusion: Findings suggest that effective remedies can be found within 3 min of remedy application. Hence Auriculohomeopathy is a quick and efficient method of remedy selection in busy clinical practice.

### 4.15. Evolved ‘PCAS’ Protocol in Prostate Syndrome and Cancer

#### Johnson, S.

Introduction: Prostate Cancer Syndrome Protocol was my discovery of a new causal modality in some patients with prostate cancer/syndrome. I have evolved it further, innovated it for auricular application plus linked it to my Genomic Acupuncture. A first for an international professional audience.

#### Professor Dr. Steven Johnson Dr. Ac Evolved PCAS Guiding Protocol ©

[A] Etiology Leading into Diagnosis in Cellular E-Energy Function Model
  1. Physical trauma can cause cancer.
    MA shuffman md autumn issue 2004 college of forensic examiners.
  2. ‘E-T’ energy trauma on macro-micro level.
  3. My new discovery. Micro ‘E-T’ energy trauma of the auricular body.
  Both can cause energy blockage syndrome and cancer. In= CTM Bioscience Chinese Medicine.
[B] Causal Considerations in Examination/Diagnosis
  1. My new discovery. Poor fitting hearing devices.
  2. Visible, palpable, E-measurable ‘E-T’ trauma, burn, piercing, implant, impinge, bite.
  3. Auditory testing.
  4. Age.
  5. Auricular E-Energy reading, E-Embryo models.
[C] 5 Point PCAS Guiding Protocol
  1. New causal modality.
  2. Link ‘E-T’ markers.
  3. Innovated combined screening procedures.
  4. Earlier testing E-Markers for prostate dysfunction detection.
  5. New combined macro-micro treatment protocols.
[D] Specialist Auricular Protocols
  1. Massage, probotic, seed, moxibustion, needle, other.
  2. San Bao; Macro-Micro Acupuncture.
  3. Long Bone, E-Systems.
[E] Outcomes
  1. See Success Rate Above.
  2. Prevents a percentage of prostate cancer/syndromes developing.
  3. TReatment Option alongside Western Medicine treatment.
  4. Adaptable Protocol for Other Cancers.

### 4.16. A Demonstration of The Techniques Used for Atrial Fibrillation and Other Conditions Using Laser Pen and Laser Needle Modality

#### Weber, M.

During the workshop the basic ideas and different patterns of Chinese Medicine (CM) in arterial fibrillation will be described: Heart Qi Deficiency, Heart Yang Deficiency, Heart Yin Deficiency, Heart Yin Deficiency with secondary Heart Fire, Spleen Qi Deficiency, Spleen Yang Deficiency, Kidney Yang Deficiency, Kidney Yin Deficiency, Liver Qi Stagnation, Blood Stagnation, Blood Heat.

According to these patterns treatment protocols will be presented and demonstrated by 12 channel “Light-Needle”. In addition the finding of active ear acupuncture points will be demonstrated and treatment energy doses will be given as a basic protocol and as an individualized protocol by VAS.

Most effective acupuncture points will be shown how to find and treat: Neiguan (Pericardium 6), Shenmen (Heart 7), Shaofu (Heart 8), the front Mu-Point Jiuwei (Renmai 15), Sanyinjiao (Spleen 6), Taiyuan (Lung 9), Xingyian (Liver 2). The treated auricularpoints treated were: shenmen, heart and the Bahr points Valium and Haldol. 

All patients were treated by laseracupuncture (RJ—Laserpen max. 500 mW; 810 nm/infrared). The local energy doses was given at 4.0 J/body acupuncture point and 0.5 J/ear acupuncture point. 

In addition to CM treatment the use of laser areatherapy LowLevelLaserLight (LLLT) with cluster probe (RJ—Physiolaser Olympic; Cluster Probe 516C superpulsed (directed beam) 5 × 904 nm/30 Watt) positioned under sternum directed towards heart (mimic 4-chamber view) will be demonstrated.

## 5. Posters

### 5.1. Auricular Point Therapy for Insomnia in China in Recent 10 Years

#### Chen Y.; Qi, S.; Meng X.; Zeng Y.

Abstract: To explore domestic auricular point therapy for insomnia in recent 10 years. The keywords retrieving clinical research papers include auricular point, insomnia, auricular point pressing therapy, auricular acupuncture therapy, auricular bloodletting method, needle-embedding method, auricular therapeutic apparatus. The databases are CNKI, WANGFANG, and VIP. Eighty-nine target theses were collected and analyzed. It is found that the popular methods are ear point pressing therapy with the seed of cowherb. The main ear points show Xin(CO_15_), Shenmen(TF_4_), Pizhixia(AT_4_), and other auricular points as well as body acupoints were combined based on syndrome differentiation. As for patients with pathogenic fire derived from stagnation of liver-*qi*, Gan(CO_12_), Yidan(CO_11_), and Erjian(HX_6,7i_), etc. were selected to shallow puncture [1], combined with Sishencong(EX-HN 1), Shenmen(HT 7), Sanyinjiao(SP 6), Shenshu(BL 23), and Taixi(KI 3). Also, the Chinese granules of *Jieyushugan’anshen* can be added [2]. Pi(CO_13_), Wei(CO_4_), Gan(CO_12_), Zhen(AT_3_), Chuiqian(LO_4_), which is special for panasthenia, and bloodletting Erjian(HX_6,7i_) [3] are suitable for phlegm-heat disturbing insomnia. In addition, we reinforce Shenmen(HT 7), Neiguan(PC 6), Baihui(GV 20), Anmian(Extra), and reduce Zhongwan(CV 12), Fenglong(ST 40), Neiting(ST 44). Chinese *Wendan* decoction is for the syndrome [4]. As to the syndrome of imbalance between heart-*yang* and kidney-*yin*, the acupoints are Naogan(AT_3,4i_), Shen(CO_10_), Jiaogan(AH_6a_), Chuiqian(LO_4_), and Anmian(Extra), with oral Chinese drugs preparation of *Anshen* liquid [5]. We treat patients with fire excess from *yin* deficiency with Jiaogan(AH_6a_), Shen(CO_10_), Gan(CO_12_), and electroacupuncture Baihui(GV 20), Shenting(GV 24) and bilateral Anmian(Extra), Shenmen(HT 7), Neiguan(PC 6), Taixi(KI 3) and Yongquan(KI 1), or ear point pressing method combined with Chinese *Zishenqingxin* decoction [6]. Sleeplessness caused by insufficiency of heart and spleen is treated with Jiaogan(AH_6a_), Pi(CO_13_), electroacupuncture Baihui(GV 20), Shenting(GV 24) and bilateral Anmian(Extra), Shenmen(HT 7), Neiguan(PC 6), Sanyinjiao(SP 6), Zusanli(ST 36), Xinshu(BL 15), and Pishu(BL 20), Gan(CO_12_), Shen(CO_10_). Thumb-tack needle for subcutaneous embedding [7] is used at Xin(CO_15_), Shenmen(TF_4_), Pizhixia(AT_4_), Naogan(AT_3,4i_), Pi(CO_13_), Wei(CO_4_), and Chuiqian(LO_4_), plus Chinese *Guipi* decoction [8]. Regarding *qi* deficiency of heart and gallbladder insomnia, the acupoints are Yidan(CO_11_), Gan(CO_12_), and electroacupuncture Baihui(GV 20), Shenting(GV 24) and bilateral Anmian(Extra), Shenmen(HT 7), Neiguan(PC 6), Danshu(BL 19), Qiuxu(GB 40), plus Chinese *Guipi* decoction and *Chaihujialonggumuli* decoction [9]. Those combined with hypertension, auricular acupoints pressing method is beneficial for patients with heart-spleen deficiency and *qi* deficiency of heart and gallbladder [10], and the ear points refer to Neifenmi(CO_18_), Erbeigou(P_s_), Gan(CO_12_), Shen(CO_10_), Jiaogan(AH_6a_), etc. Insomnia of depression [11] can be treated with Jiaogan(AH_6a_), Shen(CO_10_), Gan(CO_12_), Yidan(CO_11_), Pi(CO_13_), Wei(CO_4_), etc.; that with type 2 diabetes [12], Jiaogan(AH_6a_), Neifenmi(CO_18_), Gan(CO_12_), Pi(CO_13_), Wei(CO_4_), Shen(CO_10_), Dachang(CO_7_), Jidian, etc. When it comes to auricular point apparatus, we often use auricular vagus nerve stimulator (TENS-200 A) at auricular concha for primary insomnia [13].

To sum up, auricular point therapy for insomnia is common, with cowherb seed pressing for the mild symptom and the combination therapy of ear point, body acupuncture and Chinese herbs for the severe.

1. Li, C.C. *Clinical Observation of Shallow Acupuncture Combined with Ear Acupoint Application for Insomnia of Pathogenic Fire Derived from Stagnation of Liver-Qi*; Fujian University of TCM: Fuzhou, China, 2016.
2. Wang, J.F. Auricular acupuncture combined with Chinese granules for insomnia subhealthy. *Mod. Health (Med. Innov. Res.*) **2008**, *5*, 152–152.
3. Chen, D.S.; Lin, Y.H. Ear point bloodletting combined with acupuncture for phlegm-heat disturbing insomnia. *J. Clin. Acupunct. Moxibustion* **2014**, *30*, 21–23.
4. Zhuo, C.Q.; Zhou, W.G.; Jiang, Q.Y. Ear point plus wendan decoction for phlegm-heat disturbing insomnia. *Guangming J. Chin. Med*. **2016**, *31*, 2817–2819.
5. Liu, Y.; Yu, T.; Zhao, Y. Sixty-four cases of insomnia Chinese herb plus auricular point for insomnia caused by imbalance between heart-*yang* and kidney-*yin*. *J. China Tradit. Chin. Med. Inf*. **2010**, *2*, 291–292.
6. Niu, G.Y.; Xu, H.X. *Zishenqingxin* decoction combined with ear point pressing method for 30 climacteric insomnia patients with fire excess from *yin* deficiency. *Guangming J. Chin. Med*. **2015**, *30*, 1441–1442.
7. Chang, J.Y.; Han, S.L. Thumb-tack needle for sleeplessness caused by insufficiency of heart and spleen. *Chin. J. Hosp. Pharm.* **2016**, *36*, 79.
8. Su, Z.G.; Li, Y.P. *Guipi* decoction plus ear acupuncture for sleeplessness caused by insufficiency of heart and spleen. *Chin. J. Med. Guide* **2012**, *14*, 78–84.
9. Zeng, Q.M.; Hu, Q.H. Twenty-five cases of *qi* deficiency of heart and gallbladder insomnia treated with Chinese *Guipi* decoction and *Chaihujialonggumuli* decoction. *J. Gansu Univ. Chin. Med*. **2016**, *33*, 56–58.
10. Du, X.J.; Sun, M.K. Ear acupoint pressing for hypertension of insomnia with different types. *Cardiovasc. Dis. J. Integr. Tradit. Chin. West. Med.* **2016**, *4*, 152–153.
11. Yang, F. Ear acupoint pressing for depression of insomnia. *Clin. Study* **2016**, *24*, 86–86.
12. Tang, Y.Q.; Fei, Y.Y.; Lu, J.J. Acupoint application plus ear acupoint pressing for type 2 diabetes of insomnia. *Nurs. Integr. Tradit. Chin. West. Med*. **2016**, *2*, 63–65.
13. Luo, M.; Qu, X.X.; Li, S.Y. Transcutaneous vagus nerve stimulation for primary insomnia and affective disorder: A report of 35 cases. *Chin. Acupunct. Moxibustion* **2017**, *37*, 269–273.

### 5.2. The Mechanism of Auricular Electroacupuncture Effect on Biliary System Dysfunction and Pain

#### Zhai, X.

Cholecystitis is a high incidence of biliary tree diseases worldwide. It has been proved that acupuncture including auricular point and body point can effectively regulate the motor dysfunction or pain of extrahepatic biliary system caused by cholecystitis. It has good curative effect on cholecystitis and some pain, but the mechanism is not clear. 

The test will use the methods of electrophysiology, ELISA, immunohistochemistry to study the effect of auricular electroacupuncture (AE) on normal and pathological changes guinea pig model. So that, before and after AE treatment, the Oddi sphincter EMG, vagus nerve potentials of gallbladder pressure, bile flow changes will be observed, so as to the comparison of hormones and neurotransmitter changes, and the initiating mechanism analysis of AE regulating reaction system of motor function bile duct and its pain. We will reveal the reaction of AE on motor function and biliary pain effect and its mechanism from the nerve-body fluid regulating, then provide a basis for the theory of meridians and viscera, and also provide a scientific basis for the prevention and treatment of biliary diseases [1].

1. Zhai, X.; Sun, C.; Rong, P.; Li, S.; McCabe, M.F.; Wang, X.; Mao, J.; Wang, S. A Correlative Relationship Between Chronic Pain and Insulin Resistance in Zucker Fatty Rats: Role of Downregulation of Insulin Receptors. *J. Pain*. **2016**, *17*, 404–413. doi:10.1016/j.jpain.2015.12.003.

### 5.3. The Similarities between the World Federation of Acupuncture-Moxibustion Societies Standard of Auricular Acupuncture Points and European System of Auriculotherapy Points according to Nogier and Bahr

#### Zhou, L.

Background: Basic and clinical research on auricular acupuncture points (AAPs) was performed in China, the United States, France and Germany. Clinical auricular acupuncture point (AAP) research was done in Italy, Austria, Switzerland, Spain, the UK, Holland, Japan, Russia, and Africa. This paper is aimed at investigating the similarities of WFAS standard of auricular acupuncture points (AAPs) and European system of AAPs according to Nogier and Bahr.

Methods: Similarities were analyzed from the perspective of name and location of auricular acupuncture points, taking the standard of the World Federation of Acupuncture-Moxibustion Societies (WFAS)-Auricular Acupuncture Point, and the European system of auricular acupuncture according to Nogier/Bahr as references.

Results: The projections of the locomotor system, including shoulder, wrist, elbow, finger, pelvis and buttock, were similar. The reflexed gastrointestinal system on the auricle, including stomach, oesophagus, duodenum, small intestine, large intestine, appendix, liver, gallbladder and pancreas were similar. The projection of the urogenital system, including ureter, urinary bladder, prostate and urethra, were similar. On the head region, only the eye point is similar. The nervous system, including temple, occiput, sub-cortex and endocrine, were similar. An additional twenty-five sub-areas or points, named by the auricular anatomical name, can be listed internationally as more widely acknowledged points.

Conclusions: There are twenty-four auricular acupuncture points sharing the same name and similar locations, and twenty-five sub-areas or points are recommended to share the auricular anatomical name with different reflexed parts of the body and different therapeutic effects [1].

1. Wang, L.; Yang, J.; Zhao, B.; Zhou, L.; Wirz-Ridolfi, A. The similarities between the World Federation of Acupuncture-Moxibustion Societies’ standards for auricular acupuncture points and the European System of Auriculotherapy Points according to Nogier and Bahr. *Eur. J. Integr. Med.* **2016**, *8*, 817–834.

### 5.4. Chinese Traditional Vaccaria Segetalis Auricular Acupoint Application (Plaster) in Tinnitus Treatment

#### Wang, Z.

Objective: To compare the difference between Vaccaria Segetalis auricular acupoint application and acupuncture.

Methods: apply at subcortex, acupoint, endocrine, acupoint, liver acupoint, kidney acupoint.

Results: Vaccaria Segetalis auricular acupoint application (plaster) therapy is better than auricular acupuncture in Tinnitus treatment.

Conclusion: Vaccaria Segetalis auricular acupoint application (plaster) therapy is better than auricular acupuncture in Tinnitus treatment, and is more convenient, safe and effective.

### 5.5. The Integration of Chinese and European Auricular Acupuncture Points Treatment of 73 Cases of Adenoid Hypertrophy in Children

#### Li, C.

Childern suffering from adenoid hypertrophy are less than 10 years old. This disease can cause the obstruction of the choanal and the pharyngeal ostium of eustachian tube, which leads to produce many kinds of diseases in the adjacent organs, Such as Secretory otitis media; sinusitis; rhinitis; bronchitis etc. It may even cause adenoid face and obstructive sleep apnea hypopnea syndrome (OSAHS). Surgery is the main treatment. The author used auricular plaster therapy to treat children with adenoid hypertrophy and achieved amazing results. According to the lesion position, selected Xiaoer Qiying pills (a Chinese pill)to put it on the Corresponding to the auriclular points, not only stimulate the auriclular points at the same time combined with drug effect, This method has immediate effect, no side effect, fast curative effect, and it is easily accepted by children.

### 5.6. The Clinical Effect of Reaction Points on Holographic-Pain-Killing Line Located in Sulcus Auriculae Posterior for Treating Patients with Pain Caused by Terminal Stage of Cancer or Post Surgery

#### Lin, L.

Background: There were many patients with pain caused by terminal stage of cancer or post surgery, which were not sensitive to morphine and other narcotics. So through decades of research, Dr. Lin applied the holographic magnetic acupuncture instrument for diagnosis and therapy, and discovered the holographic-pain-killing line.

Methods: Dr. Lin Linglan discovered the special reaction painful point at the junction of the head and ear from patients with terminal stage of cancer or anesthetic failure after the surgery, by applying her patented product, the holographic magnetic acupuncture instrument for diagnosis and therapy. And she would use acupuncture or acupoint injection with 0.5 mL lidocaine on these points, which could relieve the pain with an immediate efficacy.

Results: This therapy has already been applied widely for patients with severe pain, especially for the desperate cancer patients, which would improve their quality of life greatly. 

Conclusion: The reaction points on holographic-pain-killing line located in sulcus auriculae posterior for treating patients with pain caused by terminal stage of cancer or post surgery can be widely applied in clinic. 

### 5.7. Clinical Application of Auricular Point Treatment for Upper Respiratory Tract Infection in Children

#### Yang, L.

Background: This paper investigated application status of upper respiratory tract infection in children treated by auricular point, in order to provide references for clinical application.

Methods: The literature on auricular point treatment for upper respiratory tract infection in children was collected from CNKI and Wanfang from the start of building database to 2017. Main search terms were “auricular point”, “auricular acupuncture”, “auricular piont sticking”, “ear”, “exopathy”, “fever”, “high fever”, “common cold”, “upper respiratory tract infection”, “children”, and etc. The literature was analyzed on characteristics of point selection and treatment method of auricular point treatment for upper respiratory tract infection.

Results: Totally, 70 articles regarding upper respiratory tract infection in children treated by auricular point were collected. The categories of diseases related with auricular point treatment were bronchitis, acute tonsillitis, hepetic angina, and etc. The main auricular points selected were erjian(HX_6.7i_), vena behind ear, pizhixia(AT_4_), shenmen(TF_4_), jiaogan(AH_6a_), fei(CO_14_), qiguan(CO_16_), yanhou(TG_3_), shenshangxian(TG_2p_),and etc.; for obvious cough, add duipingjian(AT_1,2,4i_), sanjiao(CO_17_), shen(CO_10_), and etc.; for obvious constipation and wheezes bianmidian, add bianmidian (jiaowozhong(TF_3_)), dachang(CO_7_), and etc.; For asthma, add duipingjian(AT_1,2,4i_), shenmen(TF_4_), neifenmi(CO_18_), etc.; for convulsion, add shenmen(TF_4_), gan(CO_12_), zhen(AT_3_), and etc.; for recurrent respiratory tract infection and delicate children, add shen(CO_10_), pi(CO_13_), wei(CO_4_), xin(CO_15_), xiaochang(CO_6_), shierzhichang(CO_5_), neifenmi(CO_18_), and etc.; for antiadoncus, add biantaoti(LO_7,8,9_); for common code of gastrointestinal type and hepetic angina with digestive symptoms (such as abdominal pain, diarrhea), add pi(CO_13_), wei(CO_4_), dachang(CO_7_), and etc. Manipulation: ear acupiont bloodletting at erjian(HX_6.7i_) or vena behind ear, auricular piont sticking (using cowherb seed, Chinese pill), laser irradiation were commonly used. Auricular point treatment was used as adjuvant therapy for the children with severe illness status, who often received comprehensive treatment. On the base of routine western medical treatment, auricular point combined with Chinese herb (such as injectio bupleurum, lanqin oral liquid), body acupuncture, moxibustion, cupping, chiropractic, points massage, physical cooling method, and etc. were commonly used.

Conclusions: The auricular point treatment is applied widely as adjuvant therapy on the basis of drug therapy in the children of upper respiratory tract infection, which can improve the curative effect, shorten the course of disease and reduce adverse reactions.

### 5.8. Observation on Curative effect of Pinn Bloodletting Combined with Qingfei Jiedu Decoction on Acne Vulgaris Treatment

#### Wang, Y.

Background: Acne vulgaris is a chronic inflammatory skin disease of hair follicles and sebaceous glands, it is often seen in adolescence. Traditional Chinese Medicine believes that the cause was lung and stomach heat, steamed face or because the food is too spicy. Ear acupuncture therapy has the characteristics of simple operation, the patient is easy to accept, the exact effect. So the clinical application is more extensive.The pinn bloodletting has the function of clearing lung feat. Qingfei Jiedu decoction was constituted by Yinhua, Zhejiang shellfish, Scrophulariaceae and other herb. Yinhua, Zhejiang shellfish as monarch drug, with its drugs together to clear lung feat and disperse stagnation toxin. Therefore, the application of pinn bloodletting combined with Qingfei Jiedu decoction in the treatment of acne vulgaris can obtain a satisfactory effect.

Methods: We choosed 120 cases of acne vulgaris patients from Hebei College of Traditional Chinese Medicine from 2014.1–2016.12. According to different treatment methods these cases randomly divided into two groups, each group has 60 cases. The control group was treated with Qingfei Jiedu decoction. The observation group was treated with pinn bloodletting combined with Qingfei Jiedu decoction. The chi-square test was used to compare the clinical curative effect of the two groups.

Results: The total effective rate was 96.0% in the observation group and 81.0% in the control group. There was a significant difference (*p* < 0.05).

Conclusions: The application of pinn bloodletting combined with Qingfei Jiedu decoction in the treatment of acne vulgaris was significantly. It was better than Traditional Chinese Medicine oral and it should be popularized.

### 5.9. Acupuncture Can Regulate the Expression of TRPV1 and NGF in Colorectal Parts of Zymosan-Induced Model

#### Yin, Y.; Li, S.; Wang, S.

Objective: Early behavioral studies have shown that acupuncture can alleviate Zymosan-induced colorectal hypersensitivity. This study was to explore whether TRPV1 is involved in acupuncture to alleviate colorectal hypersensitivity through observe the correlation between the expression of TRPV1 and NGF in the colorectum and acupuncture intervention.

Methods: Male C57BL/6 mice were used to induce visceral hypersensitivity model, and normal saline was injected into the control group. Experiment set up six groups: hypersensitivity model group (Z + C group), hypersensitivity model acupuncture preconditioning group (Z + A1 group), hypersensitivity model acupuncture group (Z + A2 group), saline injection group (S + C group), Saline injection acupuncture preconditioning group (S + A1 group), saline injection acupuncture group (S + A2 group). Western blot was used to detect the expression of TRPV1 and NGF in the colorectum after acupuncture at classic acupoints Shangjuxu (ST37); Housanli (ST36); and the expression of double-labeled neurons TRPV1 and NGF in the spinal ganglion was detected by double immunofluorescence staining.

Results: The results of Western blot showed that the expression of TrpV1 and NGF in the colorectum in Z + C group was significantly higher than that in S + C group, and there was a significant down-regulation trend in Z + A2 group after acupuncture, While Z + A1 group had no significant change; The results of double immunofluorescence staining showed that the expression of TrpV1 and IB4 in the spinal ganglion in the Z + C group was significantly higher than that in the S + C group, and the Merged graph showed that Z + C group decreased.

Conclusion: TrpV1 in the colorectum is involved in the mechanism of acupuncture to alleviate the colorectal hypersensitivity and its effect is related to the regulation of nonpeptide neurons.

Acknowledgments: This scientific work was supported by grant from National Natural Science Foundation of China (81373724) to Shao-Jun Wang.

## 6. Author Affiliations

- Alabaster, J. Calgary foothills primary care network, Calgary, AB, Canada
- Aldridge, E. Academic Support officer, St John of God Murdoch Hospital Emergency Department, Murdoch, Western Australia
- Alimi, D. University of Pittsburgh School of Medicine, Pittsburgh, PA, USA
- Ang, T.T. Chinese Nature-Cure Institute, Singapore
- Angelo, S. Outpatient Clinic for Psychiatric and Addictive Pathology, Stanford, CA, USA
- Armstrong, K. Ocupational Therapist, Women’s Integrative Healing, St. Augustine, FL, USA
- Asis, D. Pain Management, Santa Fe, Argentina
- Aung, S. University of Alberta, Edmonton, AB, Canada
- Bahr, F. European Academy for Traditional Chinese Medicine (EATCM), Munich, Germany
- Becu, P. DVM, Lyon, France
- Bulsara, M. University of Notre Dame, WA, Australia
- Chalmers, J. Australian Acupuncture and Chinese Medicine Association, Coorparoo, Australia
- Chan, H. National addictions management service (Nams), institute of mental health, Singapore
- Chelly, J. University of Pittsburgh, Pittsburgh, PA, USA
- Chen Y. China Academy of Chinese Medical Sciences, Beijing, China
- Chen, H. The First Affiliated Hospital, Nanjing Medical University, Nanjing, China
- Chen, K.L. University of New South Wales, Kensington, NSW, Australia
- Cheng, K. Beijing University of Traditional Chinese Medicine, Beijing, China
- Chin, X.Y. National addictions management service (Nams), institute of mental health, Singapore
- Christina, F. Medical University of Graz, Graz, Austria
- Cornelia, D.M. Outpatient Clinic for Psychiatric and Addictive Pathology, Stanford, CA, USA
- Daly, M. Royal Hospital for Women, Randwick, NSW, Australia
- Deriu F. University of Sassari, Sassari, Italy
- Fan, J.J. The First Affiliated Hospital, Nanjing Medical University, Nanjing, China
- Feng, X.K. The first affiliated Hospital, Nanjing Medical University, Nanjing, China
- Flucher, C. Medical University of Graz, Graz, Austria
- Gresser, H. University Paris XI, Orsay, France
- Guo, S. National addiction management service (NAMS), institute of mental health (IMH), Singapore
- Huang, F. China Academy of Chinese Medical Sciences, Beijing, China
- Jan, A. Emergency Physician, St John of God Murdoch Hospital Emergency Department & University of Notre Dame, Perth, WA, Australia
- Johnson, S. British & International China College of Oriental Medicine, Sheffield, South Yorkshire, UK
- Kurath-Koller, S. Medical University of Graz, Graz, Austria
- Kuzulugil, A. Private Practitioner, Ankara, Turkey
- Lafitte, M. ANSYS, Inc. Holon, Israel
- Lee, C. Australian Medical Acupuncture College, Leopold, VIC, Australia
- Li, C. Private Clinic, Shen-Zhou, China
- Li, S. China Academy of Chinese Medical Sciences, Beijing, China
- Lim, R. Licensed Acupunctrist—TCM Practitioners’ Board (MOH) Spore, Laser acupuncture centre, Singapore
- Lin, L. The First Affiliated Hospital of Kunming Medical University, Kunming, China
- Lindrea, K.B. Royal Hospital for Women, Randwick, NSW, Australia
- Litscher, G. Medical University of Graz, Graz, Austria
- Liu, H. The first affiliated Hospital, Nanjing Medical University, Nanjing, China
- Liu, J.H. Guangzhou University of Chinese Medicine, Foshan, China
- Lu, J. The First Affiliated Hospital, Nanjing Medical University, Nanjing, China
- Luz, F. Acupuncturist (CMAESP), São Paulo, Brazil
- Luzina, K. Central Clinical Hospital of the Presidential Administration and Russian Railways Medical Director of TCM Department 20, Moscow, Russia
- Luzina-Chju, L. Centre for Chinese Medicine “Sin-Ya-Chju” Kalanchevskaya 17, Moscow, Russia
- Meng X. China Academy of Chinese Medical Sciences, Beijing, China
- Meng, X.N. Beijing University of Chinese Medicine, Beijing, China
- Mok, M. School of Nursing, Hong Kong Polytechnic University, Hung Hom, Kowloon, Hong Kong, China
- Nageshwar, S. ANSYS Inc., Canonsburg, PA, USA
- Niemtzow, R. Uniformed Services University of the Health Sciences, Bethesda, MD, USA
- Nogier, R. GLEM, Lyon, France
- Oei, J.L. University of New South Wales, Kensington, NSW, Australia
- Oleson, T. Auriculotherapy Certification Institute, Los Angeles, CA, USA
- Pock, A. Uniformed Services University of the Health Sciences, Bethesda, MD, USA
- Practitioner, P. Private Practitioner, Meşrutiyet Caddesi 29-19 Kızılay, Ankara, Turkey
- Qi, S. China Academy of Chinese Medical Sciences, Beijing, China
- Quah-Smith, I. Roseville Wellness Group, Roseville, NSW, Australia
- Raith, W. Medical University of Graz, Graz, Austria
- Rangon, C.M. Assistance Publique - Hôpitaux de Paris, Paris, France
- Rogers, I. St John of God Murdoch Hospital Emergency Dept & Universith of Notre Dame Australia, Fremantle, WA, Australia
- Rong, P.J. China Academy of Chinese Medical Sciences, Beijing, China
- Schindler, T. University of New South Wales, Kensington, NSW, Australia
- Schmölzer, G.M. University of Alberta, Edmonton, AB, Canada
- She, Y.F. Hebei College of Traditional chinese Medicine, Shijiazhuang, China
- Stadler, J. Medical University of Graz, Graz, Austria
- Stanton, G. Emerson Hospital, Concord, MA, USA
- Suen, L. School of Nursing, Hong Kong Polytechnic University, Hung Hom, Kowloon, Hong Kong, China
- Sun, L.N. The first affiliated Hospital, Nanjing Medical University, Nanjing, China
- Szechnyi, I. Szechenyi Health Centre, Budapest, Hungary
- Tan, J.Y. Hong Kong Polytechnic University, Hong Kong, China
- Tritschler, N. Medical University of Graz, Graz, Austria
- Urlesberger, B. Medical University of Graz, Graz, Austria
- Visser, E. University of Notre Dame, Fremantle, Western Australia
- Vulliez, C. G.L.E.M. Lyon Medical Studies Group, Lyon, France
- Wang, S. China Academy of Chinese Medical Sciences, Beijing, China
- Wang, Y. Affiliated Hospital of Hebei College of traditional Chinese Medicine, Shijiazhuang, China
- Wang, Y.Q. The First Affiliated Hospital, Nanjing Medical University, Nanjing, China
- Wang, Z. Attending Acupuncturist of Anshan City Children’s Welfare Institute, Anshan, China
- Weber, M. European TCM Laser Academy, Wierling, Senden, Germany
- Wesemann, C. Deutsche Akademie fur Akupunktur, Munich, Germany
- Wirz-Ridolfi, A. CMO Medi-China Centre for Auricular Medicine, Lasertherapy and Chinese Medicine, Reinach, Switzerland
- Wojak, W. European Academy for Traditional Chinese Medicine (EATCM), Munich, Germany
- Wu, H. Private practitioner, Bishop, CA, USA
- Xie, G.; Tan, J.Y. Hong Kong Polytechnic University, Hong Kong, China
- Yang, L. China Academy of Chinese Medical Sciences, Beijing, China
- Yin, Y. China Academy of Chinese Medical Sciences, Beijing, China
- Yoshizumi, A. Acupuncturist (CMAESP), São Paulo, Brazil
- Yu, Y.T. Institute of Acupuncture and Moxibustion, China Academy of Chinese Medical Sciences, Beijing, China
- Zeng Y. China Academy of Chinese Medical Sciences, Beijing, China
- Zhai, X. China Academy of Chinese Medical Sciences, Beijing, China
- Zhang, Z.H. The First Affiliated Hospital, Nanjing Medical University, Nanjing, China
- Zhao, B.X. Beijing University of Chinese Medicine, Beijing, China
- Zhou, L. Beijing University of Chinese Medicine, Beijing, China
- Zhu, W.J. The First Affiliated Hospital, Nanjing Medical University, Nanjing, China

